# Systems for rating bodies of evidence used in systematic reviews of air pollution exposure and reproductive and children’s health: a methodological survey

**DOI:** 10.1186/s12940-024-01069-z

**Published:** 2024-03-28

**Authors:** Sophie K. F. Michel, Aishwarya Atmakuri, Ondine S. von Ehrenstein

**Affiliations:** 1 Department of Epidemiology, Fielding School of Public Health, University of California, Los Angeles (UCLA), 650 Charles E Young Dr S, Los Angeles, CA 90095 USA; 2 Department of Molecular, Cell, and Developmental Biology, University of California, Los Angeles (UCLA), Los Angeles, CA USA; 3 Department of Community Health Sciences, Fielding School of Public Health, University of California, Los Angeles (UCLA), Los Angeles, CA USA

**Keywords:** Air pollution, Reproductive and children’s health, Evidence grading, Body of evidence, Systematic reviews, Methodological survey

## Abstract

**Background:**

Translating findings from systematic reviews assessing associations between environmental exposures and reproductive and children’s health into policy recommendations requires valid and transparent evidence grading.

**Methods:**

We aimed to evaluate systems for grading bodies of evidence used in systematic reviews of environmental exposures and reproductive/ children’s health outcomes, by conducting a methodological survey of air pollution research, comprising a comprehensive search for and assessment of all relevant systematic reviews. To evaluate the frameworks used for rating the internal validity of primary studies and for grading bodies of evidence (multiple studies), we considered whether and how specific criteria or domains were operationalized to address reproductive/children’s environmental health, e.g., whether the timing of exposure assessment was evaluated with regard to vulnerable developmental stages.

**Results:**

Eighteen out of 177 (9.8%) systematic reviews used formal systems for rating the body of evidence; 15 distinct internal validity assessment tools for primary studies, and nine different grading systems for bodies of evidence were used, with multiple modifications applied to the cited approaches. The Newcastle Ottawa Scale (NOS) and the Grading of Recommendations, Assessment, Development, and Evaluations (GRADE) framework, neither developed specifically for this field, were the most commonly used approaches for rating individual studies and bodies of evidence, respectively. Overall, the identified approaches were highly heterogeneous in both their comprehensiveness and their applicability to reproductive/children’s environmental health research.

**Conclusion:**

Establishing the wider use of more appropriate evidence grading methods is instrumental both for strengthening systematic review methodologies, and for the effective development and implementation of environmental public health policies, particularly for protecting pregnant persons and children.

**Supplementary Information:**

The online version contains supplementary material available at 10.1186/s12940-024-01069-z.

## Introduction

A range of detrimental impacts of air pollution exposure on reproductive and children’s health have been established [1–5]. However, air quality regulatory efforts, and especially those accounting for the specific vulnerabilities inherent to reproductive and children’s health, have yet to be effectively implemented on a larger scale [6–8]. Formally assessing the quality of the body of evidence, meaning the collection of available individual studies, has been identified as central to translating research into policy [9]. In fact, grading the quality of the body of evidence has become an integral part of the systematic review process [10], reflected in recent additions to the revised Preferred Reporting Items for Systematic reviews and Meta-Analyses (PRISMA) 2020 guidelines, recommending authors explicitly report their approach to the process of rating the body of evidence [11]. Evidence grading approaches were developed predominantly for clinical questions, including well-established guidelines such as the Grading of Recommendations, Assessment, Development, and Evaluations (GRADE) criteria [10, 12].

However, the field of reproductive and children’s environmental health, including research on air pollutant exposure, is affected by characteristics that may complicate the critical evaluation of primary studies and bodies of evidence:

i) The predominantly observational nature of available studies means that, due to inherent differences in study design compared to experimental studies, a different approach is required for identifying and addressing potential confounding and other biases [13–15]. Specific aspects of epidemiologic studies of air pollutant exposure and reproductive/ children’s health outcomes that may result in confounding (e.g., frequent use of spatial rather than temporal comparators, lack of covariate information from birth records or other sources), have been described [16, 17]. However, both the default ranking of experimental studies above observational studies, as well as the practice of rating primary studies based on how well they emulate a “hypothetical target RCT” have been criticized [18–23].
ii) Highly heterogeneous and dynamic population characteristics that define the field of reproductive and children’s health (e.g., vulnerabilities related to developmental stages, rapid changes in health -related behaviors) require a lifestage-specific approach. Profound physiological and developmental differences between children and adults impact the toxicity and adverse biological implications of chemical exposures, based on variations in metabolic rates, (de-)toxification processes, and vulnerability during specific developmental windows [17, 24–26].
iii) Further aspects specific to reproductive and children’s health, including generally longer expected lifespans and long latency periods, life course perspectives (e.g., developmental origins of disease), trans-generational effects, among others, necessitate a tailored approach [24].
iv) Challenges related to exposure assessments are generally an issue in observational vs. experimental studies, where exposures are not controlled by investigators, and in particular, in environmental health studies [14, 27]. Exposure assessments regarding air pollution are characterized by specific challenges (e.g., differences in the availability of air monitoring data, seasonal variations in exposure patterns, etc.) [17, 27], potentially increasing misclassification, also with regard to relevant developmental periods, such as gestational trimesters. Also, there are additional considerations with regard to reproductive and children’s health: Due to differences in body size and behaviors, among others, exposure patterns are different for developing fetuses, children, and pregnant persons vs. non-pregnant adults (e.g., relative exposure doses, exposure routes and settings, timing and duration of exposure in relation to windows of susceptibility) [24, 27–29]. For example, children have different breathing zones (due to shorter stature) and oxygen consumption patterns, affecting their individual exposure to air pollution [25].
v) The co-exposure to mixes of pollutants reflects the real-world risks faced by the global population, which may include additive/ synergistic effects between chemicals, and while modeling impacts of multiple pollutants jointly could provide more valid results, there are challenges such as collinearity and high dimensionality, among others [17, 27, 30–32].
vi) Further, the context of decision-making in environmental health research differs: Unlike in the clinical setting, environmental exposures are often assessed for risks only after exposure -often wide-spread and long-term- has already occurred in the population [28]. Also, environmental health studies are focused on protecting, rather than improving health [28]. Therefore, while clinical research is primarily concerned with demonstrating a desired treatment effect, reproductive/ children’s environmental health should, arguably, be concerned with demonstrating the *absence* of adverse effects: For the former, the burden of proof lies in demonstrating an association or effect, while for the latter, it would lie in demonstrating no association or effect, in essence, safety [33–36]. Statistical methods for testing for the absence of effects (e.g., equivalence tests) are available, and in addition to providing evidence regarding the equivalence of different exposure scenarios, may also help to reduce publication bias [37–39].

Methodological weaknesses specific to assessing evidence related to environment exposures [40, 41], and specifically ambient air pollution [42], and pregnancy outcomes [43, 44], were previously identified among systematic reviews, particularly related to assessing internal validity and a lack of transparent evidence grading methodologies. Because systematic review methodologies were primarily developed for clinical trials, their suitability for evaluating evidence from observational/ environmental health, and how these methods can best be adapted, has been debated [14]. Further, certain aspects of existing approaches, including the aforementioned default ranking of evidence from randomized controlled trials (RCT) above that from observational studies, have previously been criticized in the context of environmental health [18, 22, 23].

In this methodological survey we aimed to evaluate frameworks for critically assessing bodies of evidence, applied in systematic reviews of epidemiological studies of environmental exposures and adverse reproductive/ child health outcomes, using research on air pollution exposure as a case-study. Air pollutant exposure was chosen based on the comparability of approaches within this research area, and the large body of available systematic reviews [45]. Based on this, we exemplify and discuss challenges and recommendations for evidence grading in the context of reproductive/ children’s environmental health.

## Methods

As the unit of analysis of this work was systematic reviews, we adhered to the Preferred Reporting Items for Overviews of Reviews (PRIOR) guidelines (Supplemental Material S1, PRIOR checklist) [46], and further relevant guidance [47–51]. Two reviewers independently completed all steps of the systematic process, including screening for eligible references, extracting data, and assessing risk of bias (SM and AA). Discrepancies were resolved by discussing or by consulting with the third reviewer (OVE).

### Eligibility criteria and review selection

The inclusion criteria are presented and explained in Table 1.

**Table 1:** Inclusion criteria

**Domain**	**Inclusion criteria**	**Exclusion criteria**	**Elaboration**
Population	Human studies Reproductive and child health was defined as any timepoint from conception until 18 years of age, including studies of fertility	Reviews additionally examining other populations (i.e., unrelated adult populations) in addition that of interest were not included	This criterion was introduced to ensure the applicability and comparability of identified evidence grading approaches to studies of reproductive/ child health
Exposure	All outdoor or indoor air pollutants (PM_10_, PM_2.5_, SO_2_, NO_2_, air toxics, biofuels, etc.)	Tobacco smoke, mold, wildfires, or allergens. Reviews examining other exposures in addition to air pollution were not eligible	To maximize the applicability and comparability of identified evidence grading approaches to studies of air pollution exposure, we considered only systematic reviews focusing solely in air pollution
Outcome	All adverse reproductive or children’s health outcomes	Exposure levels, individual behaviors (e.g., physical activity) were not eligible endpoints	To ensure the comparability of systematic reviews, only those with an adverse health outcome as a primary endpoint were included
Review design	Systematic reviews of observational studies. Only reviews considered “systematic”, based on the following reporting criteria were included [52]: (i) review question (ii) reproducible search strategy (e.g., naming of databases and search platforms/engines, search dates and complete search strategy) (iii) inclusion and exclusion criteria (iv) selection (screening) methods (v) critically appraises and reports the quality/risk of bias of the included studies (vi) information about data analysis and synthesis that allows the reproducibility of the results (vii) critically appraises and reports the quality of the body of evidence	Non-systematic literature reviews, scoping reviews, pooled studies, and meta-analyses not based on formal systematic review	Non-systematic reviews may be highly dissimilar in their objectives and methods compared to systematic reviews
System for rating the body of evidence	Systematic reviews that explicitly used a published tool or framework to rate the quality of the body of evidence	Systematic reviews that did not explicitly use a tool, framework, or other published guidance for rating the body of evidence	For purposes of comparability, systematic reviews that did not rate the quality of the body of evidence, or that rated the quality of the body of evidence only as part of their discussion or in another informal manner were not included
Timeframe	Articles published from 1995 onward were considered,	Published to any of the included sources before 1995	Evidence rating was initially proposed as a stage of research synthesis in 1995 [10, 53]
Language	English or German	All other languages	While systematic reviews in other languages may be relevant, we were not able to consider other languages due to resource constraints

*Abbreviations*: *NO*_*2*_ Nitrogen dioxide, *PM*_*2*.*5*_ Fine particulate matter, *PM*_*10*_ Coarse particulate matter, *SO*_*2*_ Sulfur dioxide

As highlighted in Table 1, we identified systematic reviews explicitly employing published criteria or guidelines for assessing or rating the quality of the body of evidence, among the collection of systematic reviews of studies of air pollutant exposure and adverse reproductive and child health outcomes.

Titles, abstracts, and full-texts of the identified publications were consecutively screened, and included in the subsequent screening step, unless there was explicit indication that the publication did not meet our inclusion criteria.

### Data sources and search strategy

For identifying systematic reviews, PubMed and Epistemonikos have been identified as the database combination with the highest inclusion rate [54], and we additionally searched the database Embase. For identifying systematic reviews, in favor of built-in filters, we developed a hedge combining searches of text words, filters, and publication types, based on current recommendations for achieving maximum sensitivity [54–57].

Controlled vocabulary terms and keywords were employed to combine the concepts “air pollution”, “childhood”, and “systematic review” (Supplemental Material S2: Full electronic search strategies). We used the PubMed Reminer tool [58], and the SearchRefiner tool from the Systematic Review Accelerator website [59], to develop and assess the sensitivity and specificity of our search strategy.

On December 9, 2020, we conducted the initial systematic search of the electronic databases, without language or publication status restrictions. All searches of electronic databases were performed by SM and updated until April 07, 2023.

In addition, supplementary searches were performed using the search engines Google and Google Scholar. Search engines are used supplementarily, as these allow limited insights into how search results are produced [60]. Further, we manually performed backward and forward citation searching.

### Data extraction

Data on systematic review characteristics were extracted using a standardized data extraction form. For extracting information pertaining to the evidence grading systems, descriptions reported in the original articles, as well as cited guidance documents and further related references (i.e., organization websites, etc.) were consulted. Also, we considered the versions of the approaches used within the identified systematic reviews, although in some cases, newer versions exist. If necessary, we attempted to contact systematic review authors to identify or clarify missing or unclear information.

### Risk of bias assessment (ROBIS)

Risk of bias in systematic reviews was evaluated using the ROBIS tool, based on 1) the appropriateness of study eligibility criteria, 2) methods for identifying and selection of studies, 3) data extraction and quality appraisal methods, and 4) appropriateness of data synthesis, and 5) overall risk of bias [61].

### Qualitative analysis/synthesis

We calculated the proportion of systematic reviews explicitly employing formal evidence grading frameworks. The main characteristics of these reviews, including both the main objectives and findings, as well as the systematic review methods, were synthesized in descriptive and tabular format. Methodological characteristics, specifically the guidelines and approaches used for grading bodies of evidence were reviewed. Notably, because approaches used for assessing a body of evidence are partially based on preceding assessments of the quality or risk of bias among primary/ individual studies, both of these types of assessments in the systematic review processes were distinctly considered herein.

With regard to individual studies, quality versus risk of bias or internal validity are related but distinct concepts, concerned with the critical assessment of individual studies. Risk of bias, refers to aspects of study design, conduct, or analysis that could give rise to systematic error in study results, and can be used synonymously with internal validity, which is the extent to which bias has been prevented through methodological aspects [62]. Study quality, on the other hand, may refer to (a) reporting quality; (b) internal validity or risk of bias; and (c) external validity or directness and applicability, among others [15]. However, while risk of bias vs. study quality assessments are truly distinct concepts, they are often interchanged or merged in research practice [63]. For this reason, in this methodological survey, these approaches were considered jointly.

The quality of/ certainty in the body of evidence, on the other hand, is assessed based on strengths and limitations of a collection of individual studies, and incorporates results from preceding risk of bias assessments, as well as aspects of directness/ applicability of the identified primary studies with regard to the review question, heterogeneity/ inconsistency across studies, the magnitude and precision of effect estimates, potential publication biases, and further criteria [12, 15]. Sometimes this step is followed by subsequent ratings regarding the strength or levels of evidence, or hazard identification, across study types, outcomes, or species [15, 64].

Certain criteria are applied differently when assessing the internal validity of individual studies versus the body of evidence. For example, while an identified risk of confounding will result in a lower internal validity score for an individual study, a body of evidence may receive a higher quality rating, if all plausible confounding “would reduce a demonstrated effect, or suggest a spurious effect when results show no effect”, as noted by multiple guidelines [64–66].

We considered characteristics of frameworks for rating risk of bias in individual studies, and for grading the body of evidence, specifically as they relate to reproductive/children’s environmental health, as discussed earlier (see Table 2).

**Table 2:** Considered characteristics regarding reproductive/children’s environmental health

**Considerations applied to risk of bias or quality**^**a**^** assessment tools for individual studies with regard to reproductive/ children’s environmental health**	**Considerations applied to systems for rating bodies of evidence with regard to reproductive/ children’s environmental health**
• Is the exposure assessment method evaluated? [14, 17, 24, 25, 27–29] • Are co-exposures assessed? [17, 27, 30, 31] • Is confounding considered? [13–17]	• Are ratings assigned based on a hierarchy of study designs (i.e., experimental vs. observational studies)? [18–23] • Are developmental stages, child physiology or behaviors, or child-specific health outcomes explicitly considered in evaluating the applicability of the evidence, heterogeneity of results, or potential confounding/ biases? [17, 24–26] • As part of the directness or other domain, was the adequacy of the timing of exposure assessment and the length of follow-up considered? [24, 28, 29, 67] • How is evidence for absence of an association assessed? [28, 33–36]

^**a**^Risk of bias and quality assessments of individual studies were considered jointly herein

## Results

### Review selection process

**Fig. 1:**
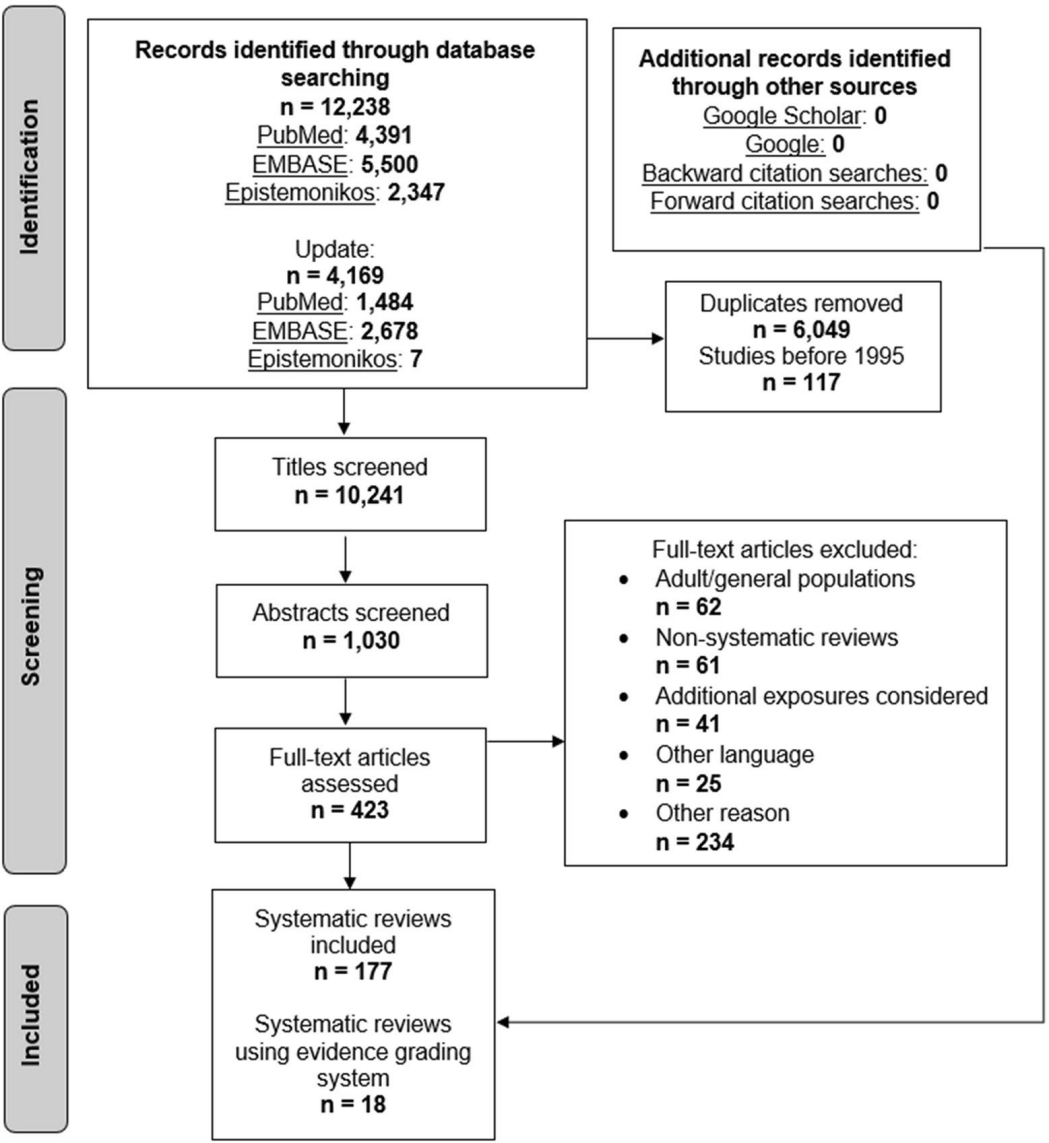
Screening process for systematic reviews

**Fig. 2:**
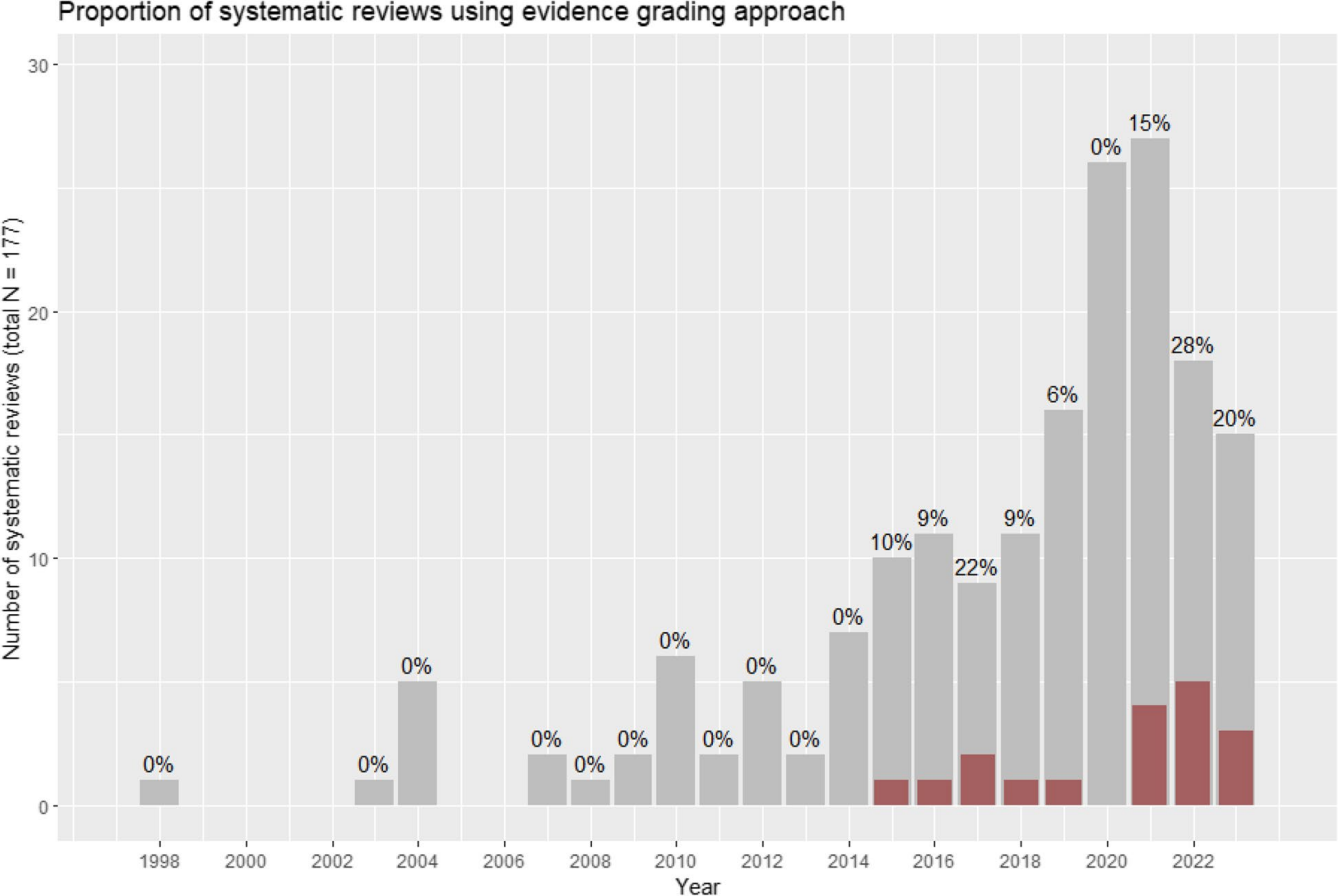
Systematic reviews per year, and proportion using evidence grading approach (designed using R software). Includes publications up until April 2023

The selection process of systematic reviews is shown in Fig. 1. After screening 10,241 titles, 1,030 abstracts and 423 full texts, 177 systematic reviews were found to assess the association between exposure to air pollution and adverse reproductive/ children’s health outcomes. The most common reasons for exclusions of full texts were that the reviews considered adult or general populations (*n* = 62), and that reviews were non-systematic (*n* = 61). Out of the 177 eligible systematic reviews, 18 articles (9.8%) explicitly reported using evidence grading systems [5, 68–84]. The proportion of systematic reviews using evidence grading systems appeared to increase over time (see Fig. 2).

### Systematic review characteristics

General characteristics of the 18 systematic reviews that used formal evidence grading systems are summarized in Table 3. These reviews were published between 2015 and 2023; and outcomes assessed were: spontaneous abortion [80], gestational diabetes mellitus [83], fetal growth [72], preterm birth [73, 79], birth weight [76], term birth weight [77], congenital anomalies [74], upper respiratory tract infections [81], bronchiolitis in infants [71], sleep-disordered breathing [70], blood pressure in children and adolescents [84], neuropsychological development [68, 78], autism spectrum disorder [5, 69], academic performance [75], or all child health outcomes [82].

**Table 3:** Characteristics of included systematic reviews (sorted by publication year)

**Author, year, synthesis type**	**PEOS of review**	**Number and type of studies included**	**Main findings**	**Risk of bias / quality assessment of individual studies**	**Risk of bias findings**	**Body of evidence assessment method**	**Body of evidence assessment findings**
Suades-González, E., et al. (2015) [68], Systematic review	**Population:** Children 0–18 years old **Exposure:** Outdoor air pollution during pregnancy, around birth or during childhood, using direct or indirect assessment methods **Outcome:** Neuropsychological development, incl. cognition, behavior, neurodevelopmental disorders, psychomotor outcomes **Studies:** Cohort, case–control, or cross-sectional design, “original research articles”. Published after 2012 in English language	20 cohort, 6 case–control, and 6 cross-sectional studies	Pre- or postnatal exposure to PAH was associated with a lower global IQ, pre- or postnatal exposure to PM_2.5_ was associated with an increased risk of ASD, and prenatal exposure to NO_x_ was associated with higher risk of ASD. All other associations showed mixed findings	Own criteria: study design, sample size, exposure and outcome assessment, confounder control	25 out of 32 included studies were rated as a “good quality studies”	Modified IARC (2006) – authors differentiate between “inadequate” and “insufficient” evidence based on whether or not studies “reported an association”	Pre- or postnatal exposure: PAH and global IQ, PM_2.5_ and ASD: *Sufficient evidence* Prenatal exposure to NO_x_ and ASD: *Limited evidence* All other associations: *Inadequate or insufficient evidence, due to few studies, low quality, or inconsistency*
Lam, J., et al. (2016) [5], Systematic review and meta-analysis	**Population:** Humans **Exposure:** Indoor/ outdoor air pollution exposure during any developmental period (maternal or paternal, in proximity to conception, during pregnancy, or childhood), prior to outcome assessment **Outcome:** Any clinical diagnosis or other continuous or dichotomous scale assessment of ASD, based on ICD 9/ 10, or DSM 4/ 5 criteria **Studies:** Original data	17 case–control studies, 4 ecological, 2 cohort studies	Per 10 μg/m^3^ increase in PM_10_: (SOR: 1.07, 95% CI: 1.06–1.08, *n* = 6 studies) and PM_2.5_ (SOR: 2.32, 95% CI: 2.15- 2.51, *n* = 3 studies) All included studies generally showed increased risk of ASD with increasing exposure to air pollution, but with some inconsistency across chemical components	Modified instrument, developed based on the Cochrane Collaboration’s tool and the AHRQ domains (i.e., selection bias, confounding, performance bias, attrition bias, detection bias, and reporting bias). Also, an approach for rating exposure assessment methods was newly developed	Majority of studies were rated as “low” or “probably low” risk of bias, besides for domains confounding and exposure assessment	Navigation Guide	*Moderate quality* of evidence across all air pollutants, due to small number of studies in meta-analysis and unexplained statistical heterogeneity
Morales-Suárez-Varela, M., et al. (2017) [69], Systematic review	**Population:** Humans **Exposure:** PM_2.5_, PM_10_, or diesel PM exposure during pregnancy/ early childhood **Outcome:** Measures of ASD symptoms or diagnosis **Studies:** Published after 2005 in English	4 cohort and 9 case–control studies	9 out of 13 studies suggested positive associations during specific exposure windows Authors conclude there is an increased risk of ASD due to PM exposure, with varying magnitude according to the particle size and composition, with the association with PM_2.5_ and diesel PM being largest	Scottish Intercollegiate Guideline Network (SIGN) used to assign levels of evidence based on study design Additional considerations were sample size, specified inclusion and assessment criteria, exposure and outcome assessment, confounder control	5 studies at a high risk of confounding or bias and a significant risk that the relationship is not causal, 8 studies at a low risk of bias and a moderate probability of causal association	SIGN levels of recommendation	*Level of recommendation: C-D* *C: A body of evidence of studies at a low risk of bias, directly applicable to the target population, with consistent results* *D: Extrapolated evidence from studies at a low risk of bias* An association between PM exposure and ASD cannot be ruled out. Data is *insufficient* to reach a consensus about ASD risk
Tenero, L., et al. (2017) [70], Systematic review	**Population:** Children **Exposure:** Indoor or outdoor air pollution **Outcome:** Sleep disordered breathing, sleep apnea, excluding asthma or SIDS **Studies:** Observational and intervention studies included, published in English language	4 cohort studies, 2 cross-sectional studies, 2 "prospective surveys” / intervention studies	Results suggest an involvement of environmental pollution in the worsening of sleep-disordered breathing in children	Centre for Evidence Based Medicine guidelines (2009, 2011)	Evidence level 3B for all studies	Centre for Evidence Based Medicine guidelines (2009, 2011)	Grade C (“cohort or case–control studies”)
King, C., et al. (2018) [71], Systematic review	**Population:** Children < 3 years old **Exposure:** Criteria air pollutants^a^, at any time period before hospitalization, categorized as acute (less than 7 days), sub-chronic (1 month prior), or lifetime exposure **Outcome:** Hospital admission, emergency department visits, unscheduled primary care visits, or critical care admission for bronchiolitis **Studies:** Cohort, case–control, time series, case-crossover designs included, ecological designs excluded	4 case–control and 4 case-crossover studies	Long term exposure to PM_2.5_, and acute exposure to SO_2_ and NO_2_ may be associated with increased risk of hospitalization for bronchiolitis. Results for other pollutants were inconsistent	NOS, in addition to considerations of specific aspects of: selection bias, exposure and outcome assessment, adjustment for confounders. Studies were rated at low risk of bias if they adjusted for at least two prespecified confounders. Further, studies were classified as higher quality if infants were < 2 years old	NOS score 7–8 (good quality) 2 studies rated as “unclear/ high” risk of bias across all domains, 6 others were rated as “low” across all domains	GRADE	*Low to moderate*, frequent downgrading for inconsistency, upgrading for precision, and up- or downgrading for study quality
Fu, L., et al. (2019) [72], Systematic review and meta-analysis	**Population:** Fetuses, infants at birth **Exposure:** Criteria air pollutants^a^ **Outcome:** Fetal growth indicators during pregnancy and anthropometric measurements at birth. Studies reporting exposure windows, sample size, and partial regression coefficient with 95% CI or standard errors were included **Studies:** English or Chinese language	11 prospective and 4 retrospective studies	Higher PM_2.5_ exposure during entire pregnancy negatively associated with head circumference at birth (-0.30 cm, 95% CI: -0.49 to -0.10), and NO_2_ exposure during entire pregnancy associated with shorter length at birth (-0.03 cm, 95% CI: -0.05 to -0.02). All other associations were not significant or inconclusive	ACROBAT-NRSI, with modifications	7 studies rated at low, 5 at moderate, and 3 at high risk of bias. Common concerns were exposure or outcome assessment methods, lack of confounder control, or study design (retrospective studies)	GRADE	PM_2.5_ and head circumference: *Moderate* PM_10_ and head circumference: *Low* NO_2_ and birth length: *Low* NO_2_ and head circumference: *Very low* Downgraded for risk of bias and inconsistency, upgraded for dose–response gradient
Rappazzo, KM., et al. (2021) [73], Systematic review and meta-analysis	**Population:** Any population capable of becoming pregnant, including those at increased risk of preterm birth **Exposure:** O_3_ exposure during 1st or 2nd trimester. Exposure assessment must have covered entire trimester. Only studies reporting a continuous exposure contrast included **Outcome:** Preterm birth **Studies:** Cohort, case–control studies. Reviews and abstract-only references excluded. Only English language articles included	16 cohort, 3 case control studies	Increased risk of PTB from exposure during 1st trimester (SOR per 10 ppb increase in O_3_: 1.06, 95 CI: 1.03–1.10), and during 2nd trimester (SOR per 10 ppb: 1.05, 95% CI: 1.02- 1.08)	Modified OHAT framework, domains included “participant selection, outcome, exposure, confounding (consideration of co-pollutants), analysis, selective reporting, sensitivity, and overall quality”	High *n* = 1, medium *n* = 9, and low *n* = 9 confidence. Common concerns included insufficient reporting or exposure assessment methods	OHAT	*Moderate* (no up- or downgrading in any domain)
Ravindra, K., et al. (2021) [74], Systematic review and meta-analysis	**Population:** Children < 5 years old. Live births, stillbirths, and terminations eligible **Exposure:** Outdoor or indoor air pollutants **Outcome:** Congenital anomalies, meaning anomalies of prenatal origin present at birth. Neurological defects, ASD, ADHD included **Studies:** Case–control, cohort, or ecological studies included. Published after 1950 in English	16 case–control, 9 cohort and 1 ecological study	N/A (due to serious concern with the data synthesis methods, results not reported here)	Risk of bias tool modified for prevalence studies from tool by Leboeuf-Yde and Lauritsen (Hoy et al. 2012), and ROBINS-E preliminary tool	Hoy et al.: Moderate (*n* = 4) to low (*n* = 22) ROBINS-E: Moderate (*n* = 11), low (*n* = 13), high (*n* = 1), unclear (*n* = 1)	Navigation Guide	NO_2_ and atrial septal defects, ventricular septal defects: *High* All other associations *Low to very low* Downgrading was due to risk of bias, imprecision, or inconsistency
Stenson, C., et al. (2021) [75], Systematic review	**Population:** Children and adolescents **Exposure:** TRAP pollutants exposure during pregnancy, childhood, or adolescence **Outcome:** Academic performance measured using standardized tests, exam results, GPA, other, (but not tests of cognitive function) **Studies:** Cross-sectional, ecological, prospective or retrospective cohort, panel and case–control studies. Only peer reviewed articles in English included. Abstract-only or conference materials excluded	7 cross-sectional designs, 2 retrospective cohort studies, and 1 longitudinal study	9 studies reported link between higher TRAP exposure and poorer student academic performance. Effect sizes were generally small	Adapted version of the OHAT tool. Key confounders and co-exposures were pre-determined, and adjustment for these was considered	Serious concerns about insufficient reporting and exposure assessments	OHAT approach. Also, an overall data assessment visualization table was developed to assess potential publication bias	*Low* Due to serious risk of bias, imprecision, and potential publication bias. 9 papers stated an absence of conflict of interest or reported funding sources which did not raise concerns
Uwak, I., et al. (2021) [76], Systematic review and meta-analysis	**Population:** Pregnant women **Exposure:** Prenatal exposure to ambient particulate air pollution (PM_2.5_, PM_10_, PM_2.5–10_) **Outcome:** Birth weight measured as a continuous variable. Studies reporting birth weight as z-scores were excluded **Studies:** N/R	51 cohort, 2 cross-sectional studies	Increased risk observed for PM_2.5_ exposure in the 2nd or 3rd trimester: -5.69 g (95% CI: − 10.58, − 0.79, I^2^: 68%) and -10.67 g birth weight (95% CI: − 20.91, − 0.43, I^2^: 84%), respectively. Over the entire pregnancy: -27.55 g (95% CI: − 48.45, − 6.65, I^2^: 94%) PM_10_ exposure in the 3rd trimester and the entire pregnancy: -6.57 g (95% CI: − 10.66, − 2.48, I^2^: 0%) and -8.65 g (95% CI: − 16.83, − 0.48, I^2^: 84%), respectively	Navigation Guide: recruitment strategy, blinding, confounding, exposure assessment, incomplete outcome data, selective outcome reporting, conflicts of interest, other concerns	Risk of bias generally “low”/ “probably low” for most domains. 43% of studies at “probably high” risk of bias for exposure assessment method (reliance on county-level monitoring data without adequate temporal coverage/ spatial resolution)	Navigation Guide	PM_2.5_: *Low*, due to imprecision and/or unexplained heterogeneity PM_10_: *Low* (imprecision) to *moderate* PM_2.5–10_: *Very low/low* (risk of bias, inconsistency, and imprecision)
Gong, C., et al. (2022) [77], Systematic review and meta-analysis	**Population:** Full-term, singleton neonates **Exposure:** Ambient PM_2.5_ **Outcome:** Change in grams of term birth weight (≥ 37 weeks of gestation) as a continuous variable **Studies:** Epidemiological studies	61 cohort, 1 case–control	Per 10 μg/m^3^ increase in PM^2.5^: − 16.54 g (95% CI: − 20.07 g, − 13.02 g, I^2^ = 96%)	NOS (only performed for studies included in meta-analysis)	Most studies (*n* = 22) obtained high NOS scores (7 + stars) Others (*n* = 9) obtained fair/low scores (5–6 stars)	GRADE (only for studies included in meta-analysis)	Overall: *Very low* (downgraded for high heterogeneity) Subgroup of studies using LUR-models: *Moderate*, upgraded for reasonable residual confounding which could reduce the effect estimates
Lin, LZ., et al. (2022) [78], Systematic review and meta-analysis	**Population:** Children 0–18 years old **Exposure:** Ambient PM exposure (pre-conception, prenatal, postnatal) **Outcome:** Neurodevelopmental disorders (ASD, ADHD, dyslexia, etc.). Confirmed by clinician or structured interview **Studies:** Case–control, cross-sectional, cohort, case-crossover, time-series, and panel studies. Full-text must be available; in English; did not include abstracts, reviews, conference materials	16 case–control, 13 cohort, 2 cross-sectional studies	Increased risk of ASD linked to exposures to PM_2.5_ during prenatal periods (SOR: 1.32, 95%CI, 1.03–1.69), 1st year after birth (SOR: 1.62, 95%CI, 1.22–2.15) and 2nd year after birth (SOR: 3.13, 95%CI, 1.47–6.67). Inconsistent evidence found for other types of PM and neurodevelopmental outcomes Some heterogeneity explained by exposure assessment period and study country	NOS, AHRQ, and Cochrane ROB tool	NOS score for case–control and cohort studies: 6–8. AHRQ score for cross-sectional studies: moderate and low quality. Cochrane ROB tool for all studies: 25 studies rated at low risk of bias, 5 as unclear, 1 as high	The Best Evidence Synthesis (BES) System for synthesis without meta-analysis. GRADE for meta-analytical results	BES: PM_2.5_ exposure and the risk of ASD in 1st year after birth: *Strong evidence* PM_2.5_ exposure and the risk of ASD in 3rd year after birth: *Moderate evidence* 63 other associations: *insufficient or no evidence* GRADE: PM_2.5_ exposure and ASD in 2nd year after birth: *Moderate* (upgraded for magnitude of effect), 12 other associations: *low* (*n* = 3) or *very low* (*n* = 9) (due to heterogeneity)
Yu, Z., et al. (2022) [79], Systematic review and meta-analysis	**Population:** Pregnant women without specific medical conditions **Exposure:** Ambient PM, short- and long-term exposures. Studies using proxies for exposure (e.g., traffic density) excluded **Outcome:** PTB diagnosed by clinical standardization or definite medical records **Studies:** Case–control, cross-sectional, time-series, or cohort studies. Excluded articles without full-text	84 case–control and cohort studies	Long-term exposure to PM_2.5_ and PM_10_ during entire pregnancy (SOR per 10 μg/m^3^: 1.084 (95% CI: 1.055–1.113) and 1.034 (95% CI: 1.018–1.049), respectively. Positive associations were also found between PM_2.5_ exposure in 2nd trimester and PTB subtypes Short-term exposure to PM_2.5_ (SOR per 10 μg/m^3^: 1.003 (1.001–1.004, I2: 65%) and 1.003 (1.001–1.005, I2: 77%) and PM_10_ (SOR: 1.001 (95% CI: 1.000–1.001). PM_10_ exposure in 2 weeks prior to birth also increased PTB risk	Risk of bias: Tailored OHAT approach Quality: NOS and Mustafic et al. (2012) for case-crossover and time-series studies	Most studies found to have moderate to high quality. Concerns were related to exposure assessment, confounding, and exclusion bias (missing data)	Navigation Guide	Moderate (Concerns regarding inconsistency, but still reached an overall rating of “moderate”)
Zhu, W., et al. (2022) [80], Systematic review and meta-analysis	**Population:** Pregnant women that conceived naturally (no IVF/ ET) **Exposure:** Chronic PM exposure (preconception to prenatal) **Outcome:** Spontaneous abortion, defined as a loss of fetus within 180 days gestation **Studies:** Only English language articles considered. Conference abstracts and reviews not included	4 case–control/ case-crossover studies, 3 cohort studies	Increased risk after PM_2.5_ and PM_10_ exposure (SOR per 10 μg/ m^3^: 1.20 (95%CI: 1.01–1.40) and 1.09 (95%CI: 1.02–1.15), respectively	NOS	All studies were rated as “high quality” (score ≥ 7)	GRADE (GRADEpro App)	Association with PM_2.5_ and PM_10_: *Moderate*
Ziou, M., et al. (2022) [81], Systematic review and meta-analysis	**Population:** Children/ adolescents < 19 years old. Studies exclusively conducted in asthmatic, allergic, or immunocompromised populations excluded **Exposure:** Ambient PM (short- or long-term exposure) **Outcome:** Upper respiratory tract infections, including otitis **Studies:** English, French, Spanish language articles from peer-reviewed journals included. Abstracts, reviews, case studies excluded	19 time-series, 4 cohort, 4 case-crossover, 4 meta-analyses, 2 cross-sectional, and 1 interventional study	Both PM_2.5_ and PM_10_ associated with hospital presentations for URTIs (SRR: 1.010, 95%CI: 1.007–1.014), SRR: 1.016, 95%CI: 1.011–1.021) in meta-analyses. Narrative analysis found total suspended particulates to be associated with URTIs, but mixed results were found for PM_2.5_ and PM_10_	Quality of studies: NOS for cohort studies, modified NOS (Modesti et al. for cross-sectional studies, and Mustafic et al. for case-crossover/ time-series) Risk of bias: Mixture of modified OHAT tool and Navigation Guide, as reported by previous reviews	Quality and risk of bias ratings ranged from low to high. Concerns included outcome and exposure assessment methods, missing data, recall bias, confounding, and selection bias	OHAT, with modifications to address case-crossover/ time-series studies	PM_2.5_ and PM_10_: *Moderate (upgraded for confounding, as studies were conducted in hospital or ambulance settings, where only a minority of children with URTIs present. Thus, there may be a bias towards null)* Total suspended particles/ PM unspecified: *Low (no change)*
Blanc, N., et al. (2023) [82], Systematic review and meta-analysis	**Population:** Humans **Exposure:** Maternal or paternal exposure to outdoor air pollutants in the preconception period. Preconception period defined as 1 month to 1 year before conception **Outcome:** All child health outcomes **Studies:** English language articles	16 cohort and 6 case–control	Exposure to outdoor air pollutants during maternal preconception period were associated with various health outcomes, of which birth defects showed the most consistent findings Large sample sizes, however inconsistencies observed in air pollutant levels and reported associations	NOS	All studies rated as high to moderate quality	Modified GRADE (starting observational studies at low) (Morgan 2016). Available human and experimental animal data and mechanistic data additionally considered Publication bias not assessed. Study funding sources also considered	Preconception exposure to PM_10_ and PM_2.5_ and birth defects *Moderate* PM_2.5_ and birthweight: *Moderate* All other associations: *Low*
Liang, W., et al. (2023) [83], Systematic review and meta-analysis	**Population:** Pregnant women **Exposure:** Ambient air pollutants. Excluded studies assessing exposure exclusively to indoor pollutants, traffic, or extreme natural environments. Categorical comparisons not included **Outcome:** Risk of GDM, with explicit diagnostic criteria reported **Studies:** Cohort studies included. Conference abstracts and studies without accessible data excluded	15 prospective, 16 retrospective cohort studies	Exposure to NO_2_, SO_2_, PM_2.5_, PM_10_, BC, and nitrate may significantly increase the risk of GDM. PM_2.5_ exposure had largest effect on GDM risk	NOS	All studies rated as high quality (7–9 stars)	Modified GRADE for air pollution studies by WHO (starting observational studies at moderate)	NO_2_ exposure during entire pregnancy, and BC exposure in first trimester: *Low* All other pollutants and exposure periods (32 associations): *Moderate (14) to high (18)* Main reasons for downgrading were high risk of bias and wide 80% prediction intervals
Tandon, S., et al. (2023) [84], Systematic review and meta-analysis	**Population:** 10–19-year-old children/ adolescents **Exposure:** Short- or long-term exposure to ambient air pollutants **Outcome:** Blood pressure (BP) **Studies:** Longitudinal (cohort, panel) or cross-sectional studies	5 cohort, 3 cross-sectional studies	Non-significant associations were observed for cohort studies assessing long-term exposure to PM_10_, PM_2.5_, and NO_2_. Significant positive associations were observed for cross-sectional studies assessing long-term exposure to PM_10_ (0.34 mmHg, 95% CI: 0.19, 0.50) and NO_2_ on diastolic BP (0.40 mmHg, 95% CI: 0.09, 0.71), and PM_10_ on systolic (0.48 mmHg, 95% CI: 0.19, 0.77) BP	NOS for cohort studies, adapted NOS for cross-sectional studies (from Herzog et al.)	3 studies rated as good quality, 4 as fair quality, 1 as poor quality	GRADE	*Low to very low* Reasons for downgrading: risk of bias (due to potential selection bias, loss of follow-up, issues with exposure and outcome assessment methods), and inconsistency

*Abbreviations*: *ACROBAT-NRSI* A Cochrane Risk Of Bias Assessment Tool: for Non-Randomized Studies of Interventions, *ADHD* Attention deficit hyperactivity disorder, *AHRQ* Agency for Healthcare Research and Quality, *ASD* Autism spectrum disorder, *BC* Black carbon, *BES* Best Evidence Synthesis, *BP* Blood pressure, *CI* Confidence interval, *DSM* Diagnostic and Statistical Manual of Mental Disorders, *GDM* Gestational diabetes mellitus, *GPA* Grade point average, *GRADE* Grading of Recommendations, Assessment, Development, and Evaluations, *I*^*2*^ Heterogeneity statistic, *IARC* International Agency for Research on Cancer, *ICD* International classification of diseases, *IQ* Intelligence quotient, *IVF/ ET* In vitro fertilization or embryo transfer, *NOS* Newcastle Ottawa Scale, *NO*_*2*_ Nitrogen dioxide, *NO*_*x*_ Nitrogen oxides, *O*_*3*_ Ozone, *OHAT* Office of Health Assessment and Translation, *PAH* Polycyclic aromatic hydrocarbons, *PM* Particulate matter, *PM*_*2*.*5*_ Fine particulate matter, *PM*_*10*_ Coarse particulate matter, *PTB* preterm birth, *ROB* Risk of bias, *ROBINS-E* Risk of bias in non-randomized studies of exposure, *SIDS* Sudden infant death syndrome, *SIGN* Scottish Intercollegiate Guidelines Network, *SO*_*2*_ Sulfur dioxide, *SOR* Summary odds ratio, *SSR* Summary risk ratio, *TRAP* Traffic-related air pollution, *URTI* Upper respiratory tract infection, *WHO* World Health Organization

^a^Criteria air pollutants: PM_10_, PM_2.5_, NO_2_, SO_2_, O_3_, CO, lead

None of the included reviews specified inclusion criteria related to the method of exposure assessment (e.g., modeling vs. monitoring approaches) (see Table 3). Two reviews considered both intervention and observational studies [70, 81], while the others included only observational studies. Between 7 and 84 studies were included by the individual reviews (Table 3) [79, 80]. One review included only studies using air monitoring stations’ data [74], while others reported a variety of exposure assessment methods and data sources. Individual-level measures of exposure (e.g., adducts in cord blood, backpack for individual monitoring) were reported for few studies included in the systematic reviews [68, 72, 76, 77].

The majority of systematic reviews included fixed or random effects meta-analyses, while five refrained for statistical pooling and synthesized their findings in narrative form [68–71, 75]. All meta-analyses included adjusted effect estimates; several reported only considering single-pollutant models.

### ROBIS assessment results

**Fig. 3:**
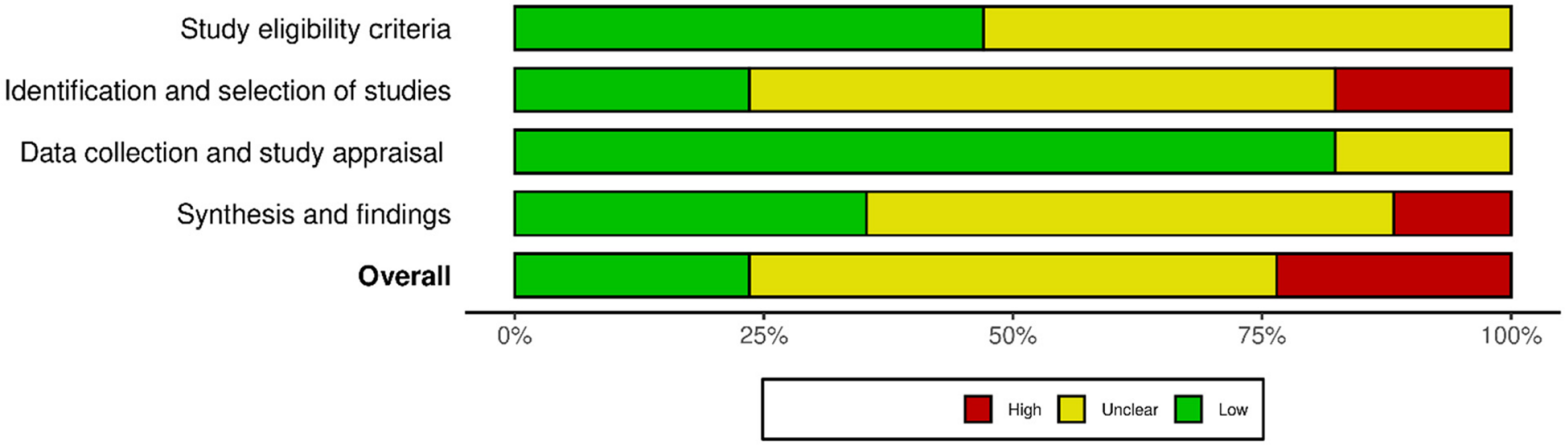
Summary of risk of bias assessment. Designed using the *robvis* tool [85]

Four of the included systematic reviews were rated at a low risk of bias [5, 71, 75, 76], four at a high risk of bias [68–70, 74], and the remaining ten at an unclear risk of bias. The most critical concerns related to methods used to search for primary studies, synthesis approaches, and insufficient reporting (Fig. 3). Between one and eight databases were searched by the various review teams [69, 75]. Six groups made no additional efforts to identify published or unpublished literature [68, 69, 71, 79, 81, 82], while eight additionally screened the reference lists of included studies and/ or those of relevant reviews [70, 72, 74–76, 80, 83, 84], in some cases additionally searching relevant reports [73], using web search engines [78], and one further searched grey literature databases and relevant websites, performed forward citation searches, and contacted experts in the field (Supplemental Materials S3 and S4: Details of ROBIS assessment) [5]. Methods used for primary study appraisal, synthesis, and evidence grading are described further below.

### Methodological characteristics- Methods for assessing risk of bias/quality in primary studies

The 18 included systematic reviews used 15 distinct approaches for assessing risk of bias/ quality/ internal validity among primary studies (Tables 3 and 4). The Newcastle–Ottawa Scale (NOS) was the most commonly cited tool (*n* = 9 reviews) [86], with an additional four reviews using modified NOS versions, followed by the Office of Health Assessment and Translation (OHAT) approach (*n* = 4 reviews)[66]. However, multiple reviews reported using multiple tools, in order to assess quality and risk of bias separately, as well as to address various study designs (e.g., cohort vs. cross-sectional studies) included within reviews [74, 78, 79, 81, 84]. Further, six reviews modified/ tailored the selected tools themselves [69, 71–73, 75, 81], while four reviews used tools as modified by preceding systematic reviews [74, 79, 81, 84].

**Table 4:** Risk of bias/ quality assessment tools for primary studies used by included systematic reviews

**Tool used**	**Number of reviews using approach**	**Number of reviews that used modifications**	**Originally developed for**	**Exposure assessment (original version)**	**Co-exposures (original version)**	**Confounding (original version)**
Newcastle–Ottawa Scale (NOS) [86]	9 [71, 77–84]	2 [71, 81]	Evaluating non-randomized studies in systematic reviews	Yes (general)^a^	No	Yes
Mustafic et al. (modified NOS) [87]	2 [79, 81]	0	Time-series and case-crossover studies of air pollution exposure	Yes	Possible (“long-term trends”)	Yes
Herzog et al. (modified NOS) [88]	1 [84]	0	Cross-sectional studies (developed for vaccine-related knowledge, attitude, and behavior)	Yes (general)^a^	No	Yes
Modesti et al. (modified NOS) [89]	1 [81]	0	Cross-sectional studies (developed for studies of blood pressure)	Yes (general)^a^	No	Yes
Office of Health Assessment and Translation (OHAT) [15, 66, 90]	4 [73, 75, 79, 81]	3 [73, 75, 81]	Systematic reviews and evidence integrations of environmental health research	Yes	Yes	Yes
Navigation Guide [5, 64]	2 [76, 81]	0	Systematic reviews of human studies in environmental health	Yes	No^§^	Yes
ACROBAT-NRSI [91] (later ROBINS-I) (97)	1 [72]	1 [72]	Non-randomized studies of interventions	Yes (“measurement of intervention”)	Yes (“co-interventions”)	Yes
Agency for Healthcare Research and Quality (AHRQ) [62]	1 [78]	0	Systematic reviews of studies of healthcare interventions	Yes (general)^a^	Yes (“co-interventions”)	Yes
Cochrane tool for RCTs (ROB 1) [92]	1 [78]	0	Systematic reviews of individual RCTs	No	No	Yes (randomization)
Hoy et al. [93]	1 [74]	0	Prevalence studies of low back and neck pain	No	No	No
ROBINS-E (preliminary version) [94]	1 [74]	0	Non-randomized studies of exposures	Yes	Yes	Yes
Centre for Evidence Based-Medicine (CEBM) [95, 96]	1 [70]	Unclear (insufficiently reported)	Clinical decision-making	Yes (general)^a^	No	Yes
Scottish Intercollegiate Guidelines Network (SIGN) [97]	1 [69]	1 [69]	Guideline development of clinical care (all study designs)	Yes (general)^a^	No	Yes
Own criteria [5, 68]	2 [5, 68]	N/A	N/A	Yes	No	Yes

*Abbreviations*: *ACROBAT-NRSI* A Cochrane Risk Of Bias Assessment Tool: for Non-Randomized Studies of Interventions, *AHRQ* Agency for Healthcare Research and Quality, *CEBM* Centre for Evidence Based-Medicine, *NOS* Newcastle Ottawa Scale, *OHAT* Office of Health Assessment and Translation, *ROB* Risk of bias, *ROBINS-E* Risk of bias in non-randomized studies of exposure, *ROBINS-I* Risk of bias in non-randomized studies of interventions, *SIGN* Scottish Intercollegiate Guidelines Network

^a^Yes (general) refers to tools that include a criterion relating to the validity of the exposure assessment method, without clear relevance to environmental exposures

^§^In other case studies (e.g., on flame retardant exposure) other pollutants were considered under the confounding domain, but not in the version considered herein

The tools originated from a wide range of research fields (see Table 4), and only the Navigation Guide and OHAT approaches, used by six reviews, were developed specifically for environmental health research [64, 66]. The Risk of Bias in Non-randomized Studies of Exposure (ROBINS-E) tool was developed for studies of non-randomized studies of exposures and used in one review in its preliminary version [94]. Two reviews newly developed their own criteria for assessing primary study quality/ risk of bias [5, 68]. Notably, Lam et al. further developed the Navigation Guide risk of bias tool with expert input, as part of their application of the Navigation Guide methodology. This included developing an approach for rating exposure assessment methods for different air pollutants/ chemical classes [5]. This approach was subsequently adopted by other identified reviews [76, 81].

Exposure assessment methods in general were evaluated in all but two out of fifteen approaches [92, 93], although we considered only four tools applicable to environmental/ air pollution exposures in this regard [64, 66, 87, 94]. Co-exposures were explicitly considered by five tools [15, 62, 87, 91, 94], while all but one tool assessed confounder control [93]. However, review authors modified existing tools in some cases, for example adding considerations of sample size, selection bias, exposure assessment method, and confounder adjustment [69, 71]. Another review group used subgroup analyses to explore the effect of different exposure assessment methods [77].

### Methodological characteristics- Evidence assessment methods

As stated above, 18 out of 177 systematic reviews used formal systems for assessing the quality/certainty of the body of evidence, and nine different approaches were used f by these 18 reviews (see Table 5), including published modifications of existing tools. The majority of reviews (*n* = 8) used the GRADE system [12], followed by modified versions of GRADE, namely the Navigation Guide (*n* = 4) [64], an approach developed by the World Health Organization (WHO) for air pollution research (*n* = 1) [98], and a modified version for environmental health research (*n* = 1) [99]. Other approaches were adopted from OHAT (*n* = 3) [15], and the International Agency for Research on Cancer’s (IARC) preamble for monographs (2006) [100], among others (see Table 5) [95–97, 101]. Modifications to and deviations from the frameworks were noted [68, 69, 71, 81].

**Table 5:** Evidence grading tools used by included systematic reviews

**Tool used**	**Number of reviews using approach**	**Number of reviews that used published modified versions**	**Number of reviews that modified/ deviated from guidelines**	**Originally developed for**	**Initial grading based on study type**	**Main considerations**	**Reproductive/ children’s health- related considerations of directness, heterogeneity, confounding**	**Timing of exposure and outcome assessment considered?**	**Considerations of absence of effects**
GRADE [12, 98, 99, 102, 103]	8 [71, 72, 77, 78, 80, 82–84]	2 [82, 83]	1 [71]	Assessing quality of evidence in clinical practice	Yes (observational studies start at low, or moderate in modified version by WHO)	Study design, risk of bias among primary studies, inconsistency, imprecision, indirectness, publication bias, magnitude, dose–response association, residual opposing confounding (see modified versions for additional considerations) [99, 100]	Evidence in adults vs. children mentioned under “indirectness” [67] Provided child-specific examples regarding confounding	Timing of outcome assessment (general) considered under “indirectness” [67]	No explicit guidance provided
Navigation Guide [64, 104]	4 [5, 74, 76, 79]	0	0	Systematic reviews in environmental health	Yes (observational studies start at moderate)	Study design, risk of bias among primary studies, inconsistency, imprecision, indirectness, publication bias, magnitude, dose–response association, residual opposing confounding)	Provided child-specific examples regarding confounding	Yes, both considered as part of rating quality and strength of evidence	Yes, in terms of publication bias and in terms of “strength of evidence (i.e., evidence for no effect)”
Office of Health Assessment and Translation (OHAT) [15, 66]	3 [73, 75, 81]	0	1 [81]	Literature-based risk evaluations of environmental substances	Presence of study-design features considered (Controlled exposures, exposure prior to outcome, individual outcome data, comparison groups used)	Study-design features, risk of bias, inconsistency, imprecision, indirectness, publication bias, magnitude of effect, dose–response, residual opposing confounding, consistency (across species, populations, study designs), other	For inconsistency, the lifestage at exposure and assessment considered	Timing of exposure and outcome mentioned (in general). For inconsistency and indirectness, exposure duration and timing relative to outcome considered	Yes, in translating quality to level of evidence: “evidence of no health effect”
Preamble for monographs by the International Agency for Research on Cancer (IARC) (2006) [100]	1 [68]	0	1 [68]	Identification and evaluation of potential carcinogens (etiology)	No, but limitations of different study designs are considered in relation to research question	Assignment based on study design, quantity, and quality of included studies, statistical power, and consistency between studies Preceded by considerations including exposure assessment methods, temporal effects of exposure, use of biomarkers, criteria for causality (based on Bradford Hill criteria) [105]. In the most recent version from 2019, this is replaced by “considerations for assessing the body of epidemiological evidence” [13, 21, 105]	No	Temporal effects and latency considered prior to evidence grading	Yes (assigned for several unbiased, consistent, precise study results based on exposures covering the full expected range in humans, with adequate length of follow-up available)
Centre for Evidence-Based Medicine (CEBM) guidelines (2009, 2011) [95, 96]	1 [70]	0	0	Grading quality of evidence for use in clinical decision-making	Yes (experimental studies are rated higher than observational ones)	Study design and quality, precision, consistency, directness, magnitude of effect	No	Length of follow-up considered (in general)	No
Scottish Intercollegiate Guidelines Network (SIGN) (2011) [97]	1 [69]	0	1 [69]	Guideline development in clinical care research	Yes (observational studies cannot score higher than “B”)	Previously determined level of evidence (based on study design, risk of bias, and likelihood that “relationship is causal”), consistency of studies, and applicability of the evidence to the target population	No	No	No
The Best Evidence Synthesis (BES) System [101]	1 [78]	0	0	Clinical management guidelines for acute lower back problems	No	Number, relevance, and quality of available studies	No	No	No

*Abbreviations*: *BES* Best Evidence Synthesis, *CEBM* Centre for Evidence Based-Medicine, *GRADE* Grading of Recommendations, Assessment, Development, and Evaluations, *IARC* International agency for research on cancer, *OHAT* Office of Health Assessment and Translation, *SIGN* Scottish Intercollegiate Guidelines Network

The identified approaches for evidence grading were originally developed either for clinical practice [95–97, 101], or for research on environmental exposures [15, 64, 66, 98, 99, 104], including air pollution [98], and were characterized by highly heterogeneous methodologies. The original GRADE system assigns an initial rating based on study type, where RCTs begin at a “high” quality rating, while observational studies begin as “low”, before considering various criteria (e.g., consistency between studies), to reach a final rating of the body of evidence [106, 107]. The GRADE system as modified by the WHO (for air pollution studies) and the Navigation Guide (developed for environmental health studies, partly based on the U.S. Environmental Protection Agency’s (EPA) criteria for reproductive and developmental toxicity [28]) differ from the original version in that observational studies are initially rated as “moderate” quality, rather than “low”, among other distinguishing features (e.g., additionally calculating 80% prediction intervals to assess heterogeneity) [64, 98, 108].

While the OHAT approach is based on the GRADE system, the initial rating is based on the number of present study-design features, rather than the study type. These include: controlled exposure, exposure prior to outcome, individual outcome data, and comparison group used . Therefore, evidence from observational studies, due to a lack of controlled exposure, will never start higher than “moderate” . Unlike the GRADE system, upgrades may additionally be given for consistency across different study designs, species, or dissimilar populations, and for “other” reasons [15]. Guidance for subsequently considering quality of evidence across multiple exposures encourages considerations across the entire body of evidence .

In the IARC approach, no initial rating is assigned based on study type, although the appropriateness of different study designs in relation to the research question are considered [100]. Further criteria include study quantity and quality, statistical power, and consistency of findings. This is preceded by considerations including exposure assessment methods, temporality, use of biomarkers, and Hill’s criteria for causality [105]. In the most recent version, this is replaced by “considerations for assessing the body of epidemiological evidence” [13, 21, 105].

The Centre for Evidence Based-Medicine (CEBM) and Scottish Intercollegiate Guidelines Network (SIGN) systems again use previously assigned ratings of each included primary study, based on study type and quality, in addition to a subset of the same criteria as GRADE, but with markedly less specific guidance and explanation, compared to the aforementioned systems. The updated version of the SIGN handbook from 2019 now recommends using the GRADE system for grading evidence. The Best Evidence Synthesis (BES) system, developed for research on lower back problems, does not explicitly rate the study type as a criterion, instead presenting a highly abbreviated approach of considering merely the number, relevance, and quality of available studies [101].

In terms of considering aspects of reproductive/ children’s environmental health research in the “indirectness”, “heterogeneity”, or “confounding/ bias” domains, the Navigation Guide, GRADE approach, and OHAT framework all provide brief commentary, in the form of examples or general guidance, while the other tools make no specific reference to reproductive/ children’s health (see Table 5). Besides the SIGN and the BES systems, all tools consider the timing of exposure and/ or outcome assessment, although only the Navigation Guide and OHAT approach explicitly address this aspect with regard to reproductive/ children’s health research (e.g., developmental stages). Finally, only the Navigation Guide, OHAT approach, and IARC framework provide guidance on assessing “evidence for no effect”. Notably, systematic review authors addressed some of these aspects outside of their application of the evidence grading frameworks, in their methods (e.g., by applying relevant inclusion criteria, or by conducting subgroup analyses of different pregnancy trimesters or age groups [73, 76, 78, 83, 84]), or in their discussions.

## Discussion

This is to our knowledge the first methodological survey to systematically identify and describe evidence grading systems used in the area of air pollution exposure and adverse reproductive/ child outcomes. Of note, this is not an overview of recommended, but of practiced methods in the field. Only 18 out of 177 systematic reviews (9.8%) were found to explicitly utilize formal rating systems for bodies of evidence. Such a small proportion suggests that this process is still not common in the field, although an increase was observed after 2015 (see Fig. 2), which is in line with previous findings on evidence grading approaches used in systematic reviews of air pollution exposure [42]. The inconsistency in the approaches used—15 different risk of bias assessment and 9 different evidence grading tools used across 18 reviews- plus the numerous modifications applied, reflect a lack of consensus. The NOS and GRADE system were the most commonly used tools for assessing internal validity and for grading evidence, respectively, discussed further below. It is noteworthy that multiple reviews “borrowed” tools originating from rather unrelated fields (e.g., clinical research on lower back problems), and there was marked heterogeneity in the comprehensiveness and relevance of the employed tools.

Further, numerous systematic reviews cited preceding reviews using the same approach, in reference to their own approach [5, 74, 76–81, 83]. This suggests a “propagated” methods adoption, where systematic review authors use preceding reviews for guidance, possibly leading to the uptake of inappropriate methods [109]. This implicates that the publication of worked examples, as those provided by the Navigation Guide group [110], are essential for further improving the methodological quality of systematic reviews.

### Risk of bias assessment

Our findings indicate that systematic review authors use a wide range of approaches for assessing risk of bias/ quality among individual studies, in many cases originating from clinical or other less related fields. 13 reviews were found to use the original or a modified NOS version. The widespread use of the contested NOS may be one of the most "spectacular" examples of the risks of quotation errors and citation copying [109, 111]. Vandenberg et al. recently outlined how flawed exposure assessment methods put public health at risk [27], and this extends to a lack of appropriate and comprehensive evaluations of exposure assessment methods. The NOS includes only a cursory evaluation of exposures assessment methods that is arguably not applicable to environmental exposures. In general, risk of bias/ quality assessment tools have been criticized for focusing on mechanically determining the potential presence of biases, often based on how closely they emulate a hypothetical “target” RCT, rather than their likely direction, magnitude, and relative importance [18, 112]. Rather than assigning ratings based on study design, assessments should identify the most probable and important biases in relation to the particular population, exposure, and outcome under investigation, rate each study on how effectively it addresses each potential bias, and differences in results across studies should be considered in relation to susceptibility to each bias [14, 112–114].

The iterative development of the ROBINS-E tool [94, 115], which in its preliminary version was criticized for being based on comparisons to the “ideal” RCT, among other limitations [116], but in its final version addressed many of these concerns, including a more nuanced approach to causal inference [117], demonstrates that continuous collaboration between experts and critical appraisal of developing tools is effective and desirable. Also, the WHO has introduced a risk of bias assessment tool for air pollution exposure studies in systematic reviews [118]. In addition, informative evaluations of additional risk of bias tools available for environmental health studies have been presented [119]. Useful interactive data visualization tools exist to facilitate comparison and selection of risk of bias/ methodological quality tools for observational studies of exposures [120], collated on the basis of a preceding systematic review [63].

### Evidence grading approaches

In this methodological survey, 16 out of 18 reviews used evidence grading systems that provided higher scores to experimental (vs. non-experimental) studies or related study features. The practice of ranking evidence based on a crude hierarchy of study designs has been criticized [18–21, 23]. For one, experimental studies may be no better at reducing “intractable” confounding, and other approaches (e.g., difference-in-difference) may be much more effectual in addressing particular confounding scenarios [23]. Pluralistic approaches to causal inference, that extend beyond counterfactual and interventionist approaches, have been proposed [21, 22].

Six reviews were found to use the original GRADE system for rating bodies of evidence, for which we noted a lack of consideration with regard to heterogeneities across different developmental stages, a paucity of attention paid to the timing of exposure to environmental risks, and a lack of discussion of evidence for no association or effect, in addition to the default ranking of experimental studies above observational ones. The applicability of the GRADE approach to observational studies has previously been discussed [121, 122], and challenges with rating the body of evidence from observational studies have been reported [123–126], including rating evidence from non-randomized studies as “low” by default, difficulties in assessing complex bodies of evidence consisting of different study designs, and limited applicability regarding research on etiology, among others [124, 127].

The GRADE working group has proposed the possibility of initially rating evidence from non-randomized studies as “high”, when used in conjunction with risk of bias assessment tools like ROBINS-I [94, 115, 128, 129]. The reasoning is that the lack of randomization will usually lead to rating down by at least two levels to “low”, so ultimately, evidence from observational studies will be rated as “low” with either method [115, 129], hence, this approach is again based on the principle that non-randomized studies are inherently inferior. Other suggestions have been made to start observational studies as "moderate", as done in the Navigation Guide’s and WHO’s modified versions [64, 98], and expand criteria for upgrading [124]. In prognosis research, the GRADE system has been adapted to start observational studies at “high” [130]. Further developments of the GRADE system for environmental health research, including a recent exploration of how considerations of biological plausibility can be integrated into evidence grading [131], are in progress [99, 132].

### Reproductive and children’s environmental health: specific guidance needed

While some of the identified frameworks were found to address selected aspects, concerns persist regarding reproductive/ children's environmental health research: Firstly, the risk of bias assessment and evidence grading frameworks frequently used by existing systematic reviews often do not explicitly or comprehensively address important aspects, such as vulnerabilities related to developmental stages, considerations of exposure timing and relative dose, etc. [24, 25]. Also, only three evidence grading systems provide any guidance on assessing evidence for the absence of effects. Addressing these points would require considerations of how domains of current evidence grading frameworks are operationalized, including indirectness domains (e.g., timing of exposures, “worst-case” exposure scenarios [27], etc.), heterogeneity (disparities related to social determinants, diverse etiological mechanisms, etc.), and biases specific to research on pregnancy and childhood (e.g., live-birth bias). Some of the identified methodologies offer some insights into how existing frameworks may be adapted [66, 98, 104]: For example, considering null findings, in addition to positive ones, is advised by the Navigation Guide with regard to publication bias, meaning that an excess of null findings, especially from small or industry-sponsored studies, are also of concern [104]. With regard to subsequent assignments of levels of evidence, the OHAT approach notes that due to the intrinsic challenges of proving a negative, concluding "evidence for no effect," requires high levels of confidence in evidence. Low/ moderate confidence should be considered “inadequate evidence” for absence of effects [15].

Failing to explicitly address the defining features and major characteristics of reproductive and children’s environmental health as described above renders nonspecific tools such as the NOS and GRADE inadequate for comprehensively evaluating the unique risks posed by environmental exposures during vulnerable developmental stages and across the lifespan. Failing to account for these complexities within evidence grading frameworks may result in an incomplete understanding of the risks posed by environmental exposures during crucial developmental stages. This lack of specification may give rise to invalid assessment results both at the level of primary studies, as well at the level of bodies of evidence, and thereby lead to erroneous conclusions about the certainty of the assessed evidence. This in turn may undermine the formulation of effective policies for protecting reproductive and children’s health. Therefore, emphasizing the need for using more specialized frameworks (e.g., ROBINS-E, Navigation Guide, OHAT) for assessing studies on reproductive and children’s environmental health is paramount for ensuring accurate findings and interpretations and, ultimately, safeguarding the health of future generations. Altogether, while the addition of new tools or domains may not be needed, further consensus and published direction on how exactly these can be operationalized in the context of reproductive/ children’s environmental health may be useful. Providing explicit guidance and clear definitions, promoting the use of more applicable frameworks, and a continued refinement and tailoring of existing frameworks towards reproductive/ children’s environmental health research is critical for improving current methodologies [133].

### Further evidence grading systems and systematic review frameworks not utilized in the identified reviews

Additional evidence grading systems and systematic frameworks for environmental health research exist, but were not utilized by the identified reviews: In 2006, the EPA published a “Framework for Assessing Health Risks of Environmental Exposures to Children” [134], providing using a “lifestage” perspective. Developing specific assessment criteria during problem formulation is recommended. A weight-of-evidence approach is used, which places emphasis on higher quality studies for evidence grading [134]. Further systematic review frameworks developed for observational studies of etiology or environmental health and toxicology research include the COSMOS-E, COSTER, and SYRINA frameworks, among others. Notably, while provided guidance on evidence grading generally reflect principles of the GRADE system, specific recommendations as to what tool or approach to use [113, 135], or whether to assign an initial rating based on study type [136], are avoided.

The existence of the approaches described above, as well as those with clear relevance to reproductive/ children’s environmental health presented earlier (e.g., ROBINS-E, Navigation Guide, OHAT), together with the limited uptake we identified, suggest that the problem lies less in an absence of appropriate methods, but with their accessibility or implementation. Promoting simple, but not oversimplified, practicable, and specific guidance should be prioritized [109].

Also, calls for child-relevant extensions to the PRISMA checklist- “PRISMA-C” have been made [26, 137, 138], and are currently under development [139]. Specific recommendations regarding risk of bias and evidence assessments could be integrated herein.

### Beyond evidence grading- linking evidence and triangulation

Different types of evidence (i.e., human and non-human studies) may be combined into integrated networks of evidences within systematic reviews of environmental health risks [18, 113]. In fact, the Navigation Guide, OHAT, and IARC methodologies provide guidance on integrating evidence from human, animal, and mechanist studies [15, 64, 66, 100, 104].

Further, triangulation (i.e., leveraging differences in evidence from diverse methodological approaches with different biases to strengthen causal inference) has been encouraged for environmental health research [22, 140]. However, guidelines are needed to help researchers integrate triangulation processes into systematic reviews effectively [140].

### Implications for policy

Systematic review methods for environmental health research continue to evolve, including at the U.S. federal level, which may have a direct impact on policies to protect reproductive and children’s health: Within the EPA, revisions are being made to current systematic review methodologies [141, 142], while proposed changes to the existing “weight-of-evidence” approach, which considers a plethora of different types of evidence, in favor of a “manipulative causation” framework, are being heavily contested [18, 143, 144], and probabilistic risk-specific dose distribution analyses are being piloted, to expand beyond previous threshold-based approaches [145]. This highlights that considerations of evidence assessment methodologies span scientific, political, and legal realms, and carry massive public health implications.

We hope this work can provide a comprehensive overview of the current state of practice in the field, and serve as a starting point for those working on the further refinement or promotion of evidence grading systems for reproductive/ children’s environmental health research.

### Supplementary Information

**Additional file 1:** **Material S1.** Preferred Reporting Items for Overviews of Reviews (PRIOR) checklist. **Material S2.** Full electronic search strategies. **Material S3.** Details of ROBIS assessment: Risk of bias assessment results for each systematic review. **Material S4.** Details of ROBIS assessment: Responses to each question of the ROBIS tool.

## Data Availability

The datasets used and/or analyzed during the current study are available from the corresponding author on reasonable request.

## Abbreviations

BES: Best Evidence Synthesis
CEBM: The Centre for Evidence Based-Medicine
EPA: Environmental Protection Agency
GRADE: Grading of Recommendations, Assessment, Development, and Evaluations
IARC: International Agency for Research on Cancer’s
NOS: Newcastle Ottawa Scale
OHAT: Office of Health Assessment and Translation
PRIOR: Preferred Reporting Items for Overviews of Reviews
PRISMA: Preferred Reporting Items for Systematic reviews and Meta-Analyses
SIGN: Scottish Intercollegiate Guidelines Network
RCT: Randomized controlled trial
ROBIS: Risk of bias in systematic reviews
WHO: World Health Organization
