# Supplementary material for: Systems for rating bodies of evidence used in systematic reviews of air pollution exposure and reproductive and children’s health: a methodological survey
Source: Environ Health. 2024 Mar 28;23:32. doi: 10.1186/s12940-024-01069-z (PMC10976715; doi:10.1186/s12940-024-01069-z)
Supplement: Supplementary file 1 — Additional file 1: Material S1. Preferred Reporting Items for Overviews of Reviews (PRIOR) checklist. Material S2. Full electronic search strategies. Material S3. Details of ROBIS assessment: Risk of bias assessment results for each systematic review. Material S4. Details of ROBIS assessment: Responses to each question of the ROBIS tool. [file 12940_2024_1069_MOESM1_ESM.docx]

**Supplemental Materials**

**Title:** Systems for rating bodies of evidence used in systematic reviews of air pollution exposure and reproductive and children’s health: a methodological survey

**Authors:** Sophie K. F. Michel^1^, Aishwarya Atmakuri^2^, Ondine S. von Ehrenstein^1,3^

**Affiliations:** ^1^Department of Epidemiology, Fielding School of Public Health, University of California, Los Angeles, Los Angeles, CA, USA; ^2^Department of Molecular, Cell, and Developmental Biology, University of California, Los Angeles, Los Angeles, CA, USA; ^3^Department of Community Health Sciences, Fielding School of Public Health, University of California, Los Angeles, Los Angeles, CA, USA

**E-Mail addresses:** Sophie Michel: sophiemichel@g.ucla.edu, Aishwarya Atmakuri: aatmakuri@g.ucla.edu, Ondine S. von Ehrenstein: ovehren@ucla.edu

**ORCID:** Sophie Michel: 0000-0001-8113-7559, Aishwarya Atmakuri: 0000-0002-1648-3890, Ondine S. von Ehrenstein: 0000-0002-3366-6867

**Corresponding author:** Sophie Michel, 650 Charles E Young Dr S, Los Angeles, CA 90095, Department of Epidemiology, Fielding School of Public Health, University of California, Los Angeles, Los Angeles, CA, USA. Email: [sophiemichel@g.ucla.edu](mailto:sophiemichel@g.ucla.edu)

Material S1: PRIOR checklist

PRIOR checklist (preferred reporting items for overviews of reviews)

| Section topic | Item No | Item | Location (page) where item is reported |
| --- | --- | --- | --- |
| **Title** | | |  |
| Title | 1 | Identify the report as an overview of reviews. | 1 |
| **Abstract** | | |  |
| Abstract | 2 | Provide a comprehensive and accurate summary of the purpose, methods, and results of the overview of reviews. | 2 |
| **Introduction** | | |  |
| Rationale | 3 | Describe the rationale for conducting the overview of reviews in the context of existing knowledge. | 5-8 |
| Objectives | 4 | Provide an explicit statement of the objective(s) or question(s) addressed by the overview of reviews. | 8 |
| **Methods** | | |  |
| Eligibility criteria | 5a | Specify the inclusion and exclusion criteria for the overview of reviews. If supplemental primary studies were included, this should be stated, with a rationale. | 9-10 |
|  | 5b | Specify the definition of “systematic review” as used in the inclusion criteria for the overview of reviews. | 9-10 |
| Information sources | 6 | Specify all databases, registers, websites, organisations, reference lists, and other sources searched or consulted to identify systematic reviews and supplemental primary studies (if included). Specify the date when each source was last searched or consulted. | 10-11 |
| Search strategy | 7 | Present the full search strategies for all databases, registers and websites, such that they could be reproduced. Describe any search filters and limits applied. | 11, Supplemental Material S2 |
| Selection process | 8a | Describe the methods used to decide whether a systematic review or supplemental primary study (if included) met the inclusion criteria of the overview of reviews. | 10 |
|  | 8b | Describe how overlap in the populations, interventions, comparators, and/or outcomes of systematic reviews was identified and managed during study selection. | NA |
| Data collection process | 9a | Describe the methods used to collect data from reports. | 11 |
|  | 9b | If applicable, describe the methods used to identify and manage primary study overlap at the level of the comparison and outcome during data collection. For each outcome, specify the method used to illustrate and/or quantify the degree of primary study overlap across systematic reviews. | NA |
|  | 9c | If applicable, specify the methods used to manage discrepant data across systematic reviews during data collection. | 11 |
| Data items | 10 | List and define all variables and outcomes for which data were sought. Describe any assumptions made and/or measures taken to identify and clarify missing or unclear information. | 11, 12-13 |
| Risk of bias assessment | 11a | Describe the methods used to assess risk of bias or methodological quality of the included systematic reviews. | 11-12 |
|  | 11b | Describe the methods used to collect data on (from the systematic reviews) and/or assess the risk of bias of the primary studies included in the systematic reviews. Provide a justification for instances where flawed, incomplete, or missing assessments are identified but not reassessed. | 11-13 |
|  | 11c | Describe the methods used to assess the risk of bias of supplemental primary studies (if included). | NA |
| Synthesis methods | 12a | Describe the methods used to summarise or synthesise results and provide a rationale for the choice(s). | 12-14 |
|  | 12b | Describe any methods used to explore possible causes of heterogeneity among results. | NA |
|  | 12c | Describe any sensitivity analyses conducted to assess the robustness of the synthesised results. | NA |
| Reporting bias assessment | 13 | Describe the methods used to collect data on (from the systematic reviews) and/or assess the risk of bias due to missing results in a summary or synthesis (arising from reporting biases at the levels of the systematic reviews, primary studies, and supplemental primary studies, if included). | NA |
| Certainty assessment | 14 | Describe the methods used to collect data on (from the systematic reviews) and/or assess certainty (or confidence) in the body of evidence for an outcome. | 12-14 |
| **Results** | | |  |
| Systematic review and supplemental primary study selection | 15a | Describe the results of the search and selection process, including the number of records screened, assessed for eligibility, and included in the overview of reviews, ideally with a flow diagram. | 15-16 |
|  | 15b | Provide a list of studies that might appear to meet the inclusion criteria, but were excluded, with the main reason for exclusion. | 15 |
| Characteristics of systematic reviews and supplemental primary studies | 16 | Cite each included systematic review and supplemental primary study (if included) and present its characteristics. | 18-27 |
| Primary study overlap | 17 | Describe the extent of primary study overlap across the included systematic reviews. | NA |
| Risk of bias in systematic reviews, primary studies, and supplemental primary studies | 18a | Present assessments of risk of bias or methodological quality for each included systematic review. | 28, Supplemental Materials S3, S4 |
|  | 18b | Present assessments (collected from systematic reviews or assessed anew) of the risk of bias of the primary studies included in the systematic reviews. | 19-27 |
|  | 18c | Present assessments of the risk of bias of supplemental primary studies (if included). | NA |
| Summary or synthesis of results | 19a | For all outcomes, summarise the evidence from the systematic reviews and supplemental primary studies (if included). If meta-analyses were done, present for each the summary estimate and its precision and measures of statistical heterogeneity. If comparing groups, describe the direction of the effect. | 19-27 |
|  | 19b | If meta-analyses were done, present results of all investigations of possible causes of heterogeneity. | NA |
|  | 19c | If meta-analyses were done, present results of all sensitivity analyses conducted to assess the robustness of synthesised results. | NA |
| Reporting biases | 20 | Present assessments (collected from systematic reviews and/or assessed anew) of the risk of bias due to missing primary studies, analyses, or results in a summary or synthesis (arising from reporting biases at the levels of the systematic reviews, primary studies, and supplemental primary studies, if included) for each summary or synthesis assessed. | NA |
| Certainty of evidence | 21 | Present assessments (collected or assessed anew) of certainty (or confidence) in the body of evidence for each outcome. | 19-27 |
| **Discussion** | | |  |
| Discussion | 22a | Summarise the main findings, including any discrepancies in findings across the included systematic reviews and supplemental primary studies (if included). | 38 |
|  | 22b | Provide a general interpretation of the results in the context of other evidence. | 38-44 |
|  | 22c | Discuss any limitations of the evidence from systematic reviews, their primary studies, and supplemental primary studies (if included) included in the overview of reviews. Discuss any limitations of the overview of reviews methods used. | 38-42 |
|  | 22d | Discuss implications for practice, policy, and future research (both systematic reviews and primary research). Consider the relevance of the findings to the end users of the overview of reviews, eg, healthcare providers, policymakers, patients, among others. | 43-44 |
| **Other information** | | |  |
| Registration and protocol | 23a | Provide registration information for the overview of reviews, including register name and registration number, or state that the overview of reviews was not registered. | 4 |
|  | 23b | Indicate where the overview of reviews protocol can be accessed, or state that a protocol was not prepared. | 4 |
|  | 23c | Describe and explain any amendments to information provided at registration or in the protocol. Indicate the stage of the overview of reviews at which amendments were made. | NA |
| Support | 24 | Describe sources of financial or non-financial support for the overview of reviews, and the role of the funders or sponsors in the overview of reviews. | 3 |
| Competing interests | 25 | Declare any competing interests of the overview of reviews' authors. | 3 |
| Author information | 26a | Provide contact information for the corresponding author. | 1 |
|  | 26b | Describe the contributions of individual authors and identify the guarantor of the overview of reviews. | 3-4 |
| Availability of data and other materials | 27 | Report which of the following are available, where they can be found, and under which conditions they may be accessed: template data collection forms; data collected from included systematic reviews and supplemental primary studies; analytic code; any other materials used in the overview of reviews. | 3 |

Material S2: Full electronic search strategies

**PubMed- First systematic search**

**Researcher: Sophie Michel**

**URL:** [**https://pubmed.ncbi.nlm.nih.gov/advanced/**](https://pubmed.ncbi.nlm.nih.gov/advanced/)

**Date: 09. December 2020**

**Filters: from 1995/1/1 - current**

| # | Concept | Search string | # of hits |
| --- | --- | --- | --- |
| 1 | Exposure | Air pollution [Mesh] OR air pollutants [Mesh] OR air pollut* [tiab] OR air toxin* [tiab] OR air toxic* [tiab] OR air quality [tiab] OR particulate matter [Mesh] OR particulate matter [tiab] OR particle* [tiab] OR ultrafine [tiab] OR PM2.5 [tiab] OR PM10 [tiab] OR exhaust gas* [tiab] OR traffic-related pollution [Mesh] OR traffic-related pollut* [tiab] OR smog [tiab] OR soot [tiab] OR dust [tiab] OR ozone [Mesh] OR ozone [tiab] OR carbon monoxide [Mesh] OR carbon monoxide* [tiab] OR sulfur dioxide [Mesh] OR sulfur dioxide* [tiab] OR sulfurous anhydride* [tiab] OR nitrogen oxides [Mesh] OR nitrogen oxide* [tiab] OR nitrogen dioxide* [tiab] OR nitric oxide* [tiab] OR NO2 [tiab] OR NOx [tiab] OR polycyclic aromatic hydrocarbons [Mesh] OR polycyclic aromatic hydrocarbon* [tiab] OR polynuclear aromatic hydrocarbon* [tiab] OR radon [Mesh] OR radon [tiab] OR benzene [Mesh] OR benzene* [tiab] OR toluene [Mesh] OR toluene* [tiab] OR ethylbenzene* [tiab] OR Xylenes [Mesh] OR xylene* [tiab] OR BTEX [tiab] OR Trichloroethanes [Mesh] OR trichloroethane* [tiab] OR organic solvent* [tiab] OR chlorinated solvent* [tiab] OR indoor air pollut* [tiab] OR household air pollut* [tiab] OR wood [Mesh] OR wood* [tiab] OR stove* [tiab] OR kitchen [tiab] OR vent* [tiab] OR combust* [tiab] OR burn* [tiab] OR fuel* [tiab] OR biofuels [Mesh] OR biofuel* [tiab] OR biogas [tiab] OR biodiesel [tiab] OR kerosene [Mesh] OR kerosene [tiab] OR biomass [tiab] OR coal [Mesh] OR coal* [tiab] OR charcoal [Mesh] OR charcoal* [tiab] OR dung [tiab] | 2,274,383 |
| 2 | Population | Pregnancy [Mesh] OR pregnan* [tiab] OR gestation [tiab] OR prenatal [tiab] OR antenatal [tiab] OR embryo* [tiab] OR fetus [Mesh] OR fetus [tiab] OR fetal [tiab] OR maternal exposure [Mesh] OR maternal exposure [tiab] OR prenatal exposure delayed effects [Mesh] OR parturition [Mesh] OR parturition [tiab] OR birth* [tiab] OR preterm [tiab] OR premature [tiab] OR infant [Mesh] OR newborn [tiab] OR neonat* [tiab] OR Infant* [tiab] OR infancy [tiab] OR baby [tiab] OR babies [tiab] OR toddler* [tiab] OR child [Mesh] OR child* [tiab] OR school [tiab] OR kindergar* [tiab] OR boys [tiab] OR girls [tiab] OR nursery [tiab] OR daycare [tiab] OR teenage* [tiab] OR adolescent [Mesh] OR adolescen* [tiab] OR puberty [Mesh] OR pubert* [tiab] OR pubescen* [tiab] OR students [Mesh] OR student* [tiab] OR youth [tiab] OR pediatrics [Mesh] OR pediatric* [tiab] OR paediatric* [tiab] | 5,777,383 |
| 3 | Study type | (Systematic* [tiab] AND review* [tiab]) OR systematic review [pt] OR (Meta [tiab] OR meta- [tiab] AND analys* [tiab]) OR meta-analysis [pt] OR (evidence [tiab] AND synthes* [tiab]) OR systematicreview [Filter] OR meta-analysis [Filter] | 431,244 |
| 4 | Filter | Animals [Mesh] NOT Humans [Mesh] | 4,856,557 |
| 5 | Search | #1 AND #2 AND #3 | 4,890 |
| 6 | Search | #5 NOT #4 | 4,391 |

**PubMed- Final updated systematic search**

**Researcher: Sophie Michel**

**URL:** [**https://pubmed.ncbi.nlm.nih.gov/advanced/**](https://pubmed.ncbi.nlm.nih.gov/advanced/)

**Date: 07. April 2023**

**Filters: from 01. December 2020 - current**

| # | Concept | Search string | # of hits |
| --- | --- | --- | --- |
| 1 | Exposure | Air pollution [Mesh] OR air pollutants [Mesh] OR air pollut* [tiab] OR air toxin* [tiab] OR air toxic* [tiab] OR air quality [tiab] OR particulate matter [Mesh] OR particulate matter [tiab] OR particle* [tiab] OR ultrafine [tiab] OR PM2.5 [tiab] OR PM10 [tiab] OR exhaust gas* [tiab] OR traffic-related pollution [Mesh] OR traffic-related pollut* [tiab] OR smog [tiab] OR soot [tiab] OR dust [tiab] OR ozone [Mesh] OR ozone [tiab] OR carbon monoxide [Mesh] OR carbon monoxide* [tiab] OR sulfur dioxide [Mesh] OR sulfur dioxide* [tiab] OR sulfurous anhydride* [tiab] OR nitrogen oxides [Mesh] OR nitrogen oxide* [tiab] OR nitrogen dioxide* [tiab] OR NO2 [tiab] OR NOx [tiab] OR polycyclic aromatic hydrocarbons [Mesh] OR polycyclic aromatic hydrocarbon* [tiab] OR polynuclear aromatic hydrocarbon* [tiab] OR radon [Mesh] OR radon [tiab] OR benzene [Mesh] OR benzene* [tiab] OR toluene [Mesh] OR toluene* [tiab] OR ethylbenzene* [tiab] OR Xylenes [Mesh] OR xylene* [tiab] OR BTEX [tiab] OR Trichloroethanes [Mesh] OR trichloroethane* [tiab] OR organic solvent* [tiab] OR chlorinated solvent* [tiab] OR indoor air pollut* [tiab] OR household air pollut* [tiab] OR wood [Mesh] OR wood* [tiab] OR stove* [tiab] OR kitchen [tiab] OR vent* [tiab] OR combust* [tiab] OR burn* [tiab] OR fuel* [tiab] OR biofuels [Mesh] OR biofuel* [tiab] OR biogas [tiab] OR biodiesel [tiab] OR kerosene [Mesh] OR kerosene [tiab] OR biomass [tiab] OR coal [Mesh] OR coal* [tiab] OR charcoal [Mesh] OR charcoal* [tiab] OR dung [tiab] | 298,365 |
| 2 | Population | Pregnancy [Mesh] OR pregnan* [tiab] OR gestation [tiab] OR prenatal [tiab] OR antenatal [tiab] OR embryo* [tiab] OR fetus [Mesh] OR fetus [tiab] OR fetal [tiab] OR maternal exposure [Mesh] OR maternal exposure [tiab] OR prenatal exposure delayed effects [Mesh] OR parturition [Mesh] OR parturition [tiab] OR birth* [tiab] OR preterm [tiab] OR premature [tiab] OR infant [Mesh] OR newborn [tiab] OR neonat* [tiab] OR Infant* [tiab] OR infancy [tiab] OR baby [tiab] OR babies [tiab] OR toddler* [tiab] OR child [Mesh] OR child* [tiab] OR school [tiab] OR kindergar* [tiab] OR boys [tiab] OR girls [tiab] OR nursery [tiab] OR daycare [tiab] OR teenage* [tiab] OR adolescent [Mesh] OR adolescen* [tiab] OR puberty [Mesh] OR pubert* [tiab] OR pubescen* [tiab] OR students [Mesh] OR student* [tiab] OR youth [tiab] OR pediatrics [Mesh] OR pediatric* [tiab] OR paediatric* [tiab] | 580,915 |
| 3 | Study type | (Systematic* [tiab] AND review* [tiab]) OR systematic review [pt] OR (Meta [tiab] OR meta- [tiab] AND analys* [tiab]) OR meta-analysis [pt] OR (evidence [tiab] AND synthes* [tiab]) OR systematicreview [Filter] OR meta-analysis [Filter] | 158,203 |
| 4 | Filter | Animals [Mesh] NOT Humans [Mesh] | 277,362 |
| 5 | Search | #1 AND #2 AND #3 | 1,498 |
| 6 | Search | #5 NOT #4 | 1,484 |

**Embase- First systematic search**

**Researcher: Sophie Michel**

**URL:****https://www.embase.com/#advancedSearch/default**

**Date: 09. December 2020**

**Filters: none**

| # | Concept | Search string | # of hits |
| --- | --- | --- | --- |
| 1 | Exposure | ('Air pollution '/exp OR 'Air pollutant '/exp OR "air pollut*":ti,ab,cl,oa,kw OR "air toxin*":ti,ab,cl,oa,kw OR "air toxic*":ti,ab,cl,oa,kw OR 'air quality '/exp OR "air quality":ti,ab,cl,oa,kw OR 'particulate matter '/exp OR "particulate matter":ti,ab,cl,oa,kw OR particle*:ti,ab,cl,oa,kw OR ultrafine:ti,ab,cl,oa,kw OR PM2.5:ti,ab,cl,oa,kw OR PM10:ti,ab,cl,oa,kw OR 'exhaust gas '/exp OR "exhaust gas*":ti,ab,cl,oa,kw OR "traffic-related pollut*":ti,ab,cl,oa,kw OR smog:ti,ab,cl,oa,kw OR soot:ti,ab,cl,oa,kw OR 'dust '/exp OR dust:ti,ab,cl,oa,kw OR 'ozone '/exp OR ozone:ti,ab,cl,oa,kw OR 'carbon monoxide '/exp OR "carbon monoxide*":ti,ab,cl,oa,kw OR 'sulfur dioxide '/exp OR "sulfur dioxide*":ti,ab,cl,oa,kw OR "sulfurous anhydride*":ti,ab,cl,oa,kw OR 'nitrogen oxide '/exp OR "nitrogen oxide*":ti,ab,cl,oa,kw OR 'nitrogen dioxide '/exp OR "nitrogen dioxide*":ti,ab,cl,oa,kw OR 'nitric oxide '/exp OR "nitric oxide*":ti,ab,cl,oa,kw OR NO2:ti,ab,cl,oa,kw OR NOx:ti,ab,cl,oa,kw OR 'polycyclic aromatic hydrocarbon '/exp OR "polycyclic aromatic hydrocarbon*":ti,ab,cl,oa,kw OR "polynuclear aromatic hydrocarbon*":ti,ab,cl,oa,kw OR 'radon '/exp OR radon:ti,ab,cl,oa,kw OR 'benzene '/exp OR benzene*:ti,ab,cl,oa,kw OR 'toluene '/exp OR toluene*:ti,ab,cl,oa,kw OR 'ethylbenzene '/exp OR ethylbenzene*:ti,ab,cl,oa,kw OR 'Xylene '/exp OR xylene*:ti,ab,cl,oa,kw OR BTEX:ti,ab,cl,oa,kw OR 'Trichloroethylene '/exp OR trichloroethane*:ti,ab,cl,oa,kw OR "organic solvent*":ti,ab,cl,oa,kw OR "chlorinated solvent*":ti,ab,cl,oa,kw OR "indoor air pollut*":ti,ab,cl,oa,kw OR "household air pollut*":ti,ab,cl,oa,kw OR 'wood '/exp OR wood*:ti,ab,cl,oa,kw OR stove*:ti,ab,cl,oa,kw OR kitchen:ti,ab,cl,oa,kw OR vent*:ti,ab,cl,oa,kw OR combust*:ti,ab,cl,oa,kw OR burn*:ti,ab,cl,oa,kw OR fuel*:ti,ab,cl,oa,kw OR 'biofuels '/exp OR biofuel*:ti,ab,cl,oa,kw OR 'biogas '/exp OR biogas:ti,ab,cl,oa,kw OR 'biodiesel '/exp OR biodiesel:ti,ab,cl,oa,kw OR 'kerosene '/exp OR kerosene:ti,ab,cl,oa,kw OR biomass:ti,ab,cl,oa,kw OR 'coal '/exp OR coal*:ti,ab,cl,oa,kw OR 'charcoal '/exp OR charcoal*:ti,ab,cl,oa,kw OR dung:ti,ab,cl,oa,kw) | 2,412,264 |
| 2 | Population | ('Pregnancy '/exp OR pregnan*:ti,ab,cl,oa,kw OR gestation:ti,ab,cl,oa,kw OR 'prenatal development '/exp OR prenatal:ti,ab,cl,oa,kw OR antenatal:ti,ab,cl,oa,kw OR 'embryo '/exp OR embryo*:ti,ab,cl,oa,kw OR 'fetus '/exp OR fetus:ti,ab,cl,oa,kw OR fetal:ti,ab,cl,oa,kw OR 'maternal exposure '/exp OR "maternal exposure":ti,ab,cl,oa,kw OR 'prenatal exposure'/exp OR "prenatal exposure":ti,ab,cl,oa,kw OR 'birth '/exp OR parturition:ti,ab,cl,oa,kw OR birth*:ti,ab,cl,oa,kw OR 'prematurity '/exp OR preterm:ti,ab,cl,oa,kw OR premature:ti,ab,cl,oa,kw OR 'infant '/exp OR neonat*:ti,ab,cl,oa,kw OR newborn:ti,ab,cl,oa,kw OR Infant*:ti,ab,cl,oa,kw OR infancy:ti,ab,cl,oa,kw OR baby:ti,ab,cl,oa,kw OR babies:ti,ab,cl,oa,kw OR 'toddler '/exp OR toddler:ti,ab,cl,oa,kw OR 'child '/exp OR child*:ti,ab,cl,oa,kw OR school:ti,ab,cl,oa,kw OR kindergar*:ti,ab,cl,oa,kw OR boys:ti,ab,cl,oa,kw OR girls:ti,ab,cl,oa,kw OR nursery:ti,ab,cl,oa,kw OR daycare:ti,ab,cl,oa,kw OR teenage*:ti,ab,cl,oa,kw OR 'adolescent '/exp OR adolescen*:ti,ab,cl,oa,kw OR 'adolescence '/exp OR 'puberty '/exp OR pubert*:ti,ab,cl,oa,kw OR pubescen*:ti,ab,cl,oa,kw OR 'student '/exp OR student*:ti,ab,cl,oa,kw OR youth:ti,ab,cl,oa,kw OR 'juvenile '/exp OR juvenile:ti,ab,cl,oa,kw OR 'pediatrics '/exp OR pediatric*:ti,ab,cl,oa,kw OR paediatric*:ti,ab,cl,oa,kw) | 7,478,169 |
| 3 | Study type | [systematic review]/lim OR [meta analysis]/lim OR (systematic*:ti,ab,cl,kw AND review*:ti,ab,cl,kw) OR ((meta:ti,ab,cl,kw OR meta-:ti,ab,cl,kw) AND analys*:ti,ab,cl,kw) OR (evidence:ti,ab,cl,kw AND synthes*:ti,ab,cl,kw) | 762,670 |
| 4 | Filter | 'animal'/exp NOT 'human'/exp | 6,096,290 |
| 5 | Search | #1 AND #2 AND #3 | 5,864 |
| 6 | Search | #5 NOT #4 | 5,500 |

**Embase- Final updated systematic search**

**Researcher: Sophie Michel**

**URL:****https://www.embase.com/#advancedSearch/default**

**Date: 07. April 2023**

**Filters: 01.01.2020 onward**

| # | Concept | Search string | # of hits |
| --- | --- | --- | --- |
| 1 | Exposure | ('Air pollution '/exp OR 'Air pollutant '/exp OR "air pollut*":ti,ab,cl,oa,kw OR "air toxin*":ti,ab,cl,oa,kw OR "air toxic*":ti,ab,cl,oa,kw OR 'air quality '/exp OR "air quality":ti,ab,cl,oa,kw OR 'particulate matter '/exp OR "particulate matter":ti,ab,cl,oa,kw OR particle*:ti,ab,cl,oa,kw OR ultrafine:ti,ab,cl,oa,kw OR PM2.5:ti,ab,cl,oa,kw OR PM10:ti,ab,cl,oa,kw OR 'exhaust gas '/exp OR "exhaust gas*":ti,ab,cl,oa,kw OR "traffic-related pollut*":ti,ab,cl,oa,kw OR smog:ti,ab,cl,oa,kw OR soot:ti,ab,cl,oa,kw OR 'dust '/exp OR dust:ti,ab,cl,oa,kw OR 'ozone '/exp OR ozone:ti,ab,cl,oa,kw OR 'carbon monoxide '/exp OR "carbon monoxide*":ti,ab,cl,oa,kw OR 'sulfur dioxide '/exp OR "sulfur dioxide*":ti,ab,cl,oa,kw OR "sulfurous anhydride*":ti,ab,cl,oa,kw OR 'nitrogen oxide '/exp OR "nitrogen oxide*":ti,ab,cl,oa,kw OR 'nitrogen dioxide '/exp OR "nitrogen dioxide*":ti,ab,cl,oa,kw OR NO2:ti,ab,cl,oa,kw OR NOx:ti,ab,cl,oa,kw OR 'polycyclic aromatic hydrocarbon '/exp OR "polycyclic aromatic hydrocarbon*":ti,ab,cl,oa,kw OR "polynuclear aromatic hydrocarbon*":ti,ab,cl,oa,kw OR 'radon '/exp OR radon:ti,ab,cl,oa,kw OR 'benzene '/exp OR benzene*:ti,ab,cl,oa,kw OR 'toluene '/exp OR toluene*:ti,ab,cl,oa,kw OR 'ethylbenzene '/exp OR ethylbenzene*:ti,ab,cl,oa,kw OR 'Xylene '/exp OR xylene*:ti,ab,cl,oa,kw OR BTEX:ti,ab,cl,oa,kw OR 'Trichloroethylene '/exp OR trichloroethane*:ti,ab,cl,oa,kw OR "organic solvent*":ti,ab,cl,oa,kw OR "chlorinated solvent*":ti,ab,cl,oa,kw OR "indoor air pollut*":ti,ab,cl,oa,kw OR "household air pollut*":ti,ab,cl,oa,kw OR 'wood '/exp OR wood*:ti,ab,cl,oa,kw OR stove*:ti,ab,cl,oa,kw OR kitchen:ti,ab,cl,oa,kw OR vent*:ti,ab,cl,oa,kw OR combust*:ti,ab,cl,oa,kw OR burn*:ti,ab,cl,oa,kw OR fuel*:ti,ab,cl,oa,kw OR 'biofuels '/exp OR biofuel*:ti,ab,cl,oa,kw OR 'biogas '/exp OR biogas:ti,ab,cl,oa,kw OR 'biodiesel '/exp OR biodiesel:ti,ab,cl,oa,kw OR 'kerosene '/exp OR kerosene:ti,ab,cl,oa,kw OR biomass:ti,ab,cl,oa,kw OR 'coal '/exp OR coal*:ti,ab,cl,oa,kw OR 'charcoal '/exp OR charcoal*:ti,ab,cl,oa,kw OR dung:ti,ab,cl,oa,kw) | 440,730 |
| 2 | Population | ('Pregnancy '/exp OR pregnan*:ti,ab,cl,oa,kw OR gestation:ti,ab,cl,oa,kw OR 'prenatal development '/exp OR prenatal:ti,ab,cl,oa,kw OR antenatal:ti,ab,cl,oa,kw OR 'embryo '/exp OR embryo*:ti,ab,cl,oa,kw OR 'fetus '/exp OR fetus:ti,ab,cl,oa,kw OR fetal:ti,ab,cl,oa,kw OR 'maternal exposure '/exp OR "maternal exposure":ti,ab,cl,oa,kw OR 'prenatal exposure'/exp OR "prenatal exposure":ti,ab,cl,oa,kw OR 'birth '/exp OR parturition:ti,ab,cl,oa,kw OR birth*:ti,ab,cl,oa,kw OR 'prematurity '/exp OR preterm:ti,ab,cl,oa,kw OR premature:ti,ab,cl,oa,kw OR 'infant '/exp OR neonat*:ti,ab,cl,oa,kw OR newborn:ti,ab,cl,oa,kw OR Infant*:ti,ab,cl,oa,kw OR infancy:ti,ab,cl,oa,kw OR baby:ti,ab,cl,oa,kw OR babies:ti,ab,cl,oa,kw OR 'toddler '/exp OR toddler:ti,ab,cl,oa,kw OR 'child '/exp OR child*:ti,ab,cl,oa,kw OR school:ti,ab,cl,oa,kw OR kindergar*:ti,ab,cl,oa,kw OR boys:ti,ab,cl,oa,kw OR girls:ti,ab,cl,oa,kw OR nursery:ti,ab,cl,oa,kw OR daycare:ti,ab,cl,oa,kw OR teenage*:ti,ab,cl,oa,kw OR 'adolescent '/exp OR adolescen*:ti,ab,cl,oa,kw OR 'adolescence '/exp OR 'puberty '/exp OR pubert*:ti,ab,cl,oa,kw OR pubescen*:ti,ab,cl,oa,kw OR 'student '/exp OR student*:ti,ab,cl,oa,kw OR youth:ti,ab,cl,oa,kw OR 'juvenile '/exp OR juvenile:ti,ab,cl,oa,kw OR 'pediatrics '/exp OR pediatric*:ti,ab,cl,oa,kw OR paediatric*:ti,ab,cl,oa,kw) | 1,059,449 |
| 3 | Study type | [systematic review]/lim OR [meta analysis]/lim OR (systematic*:ti,ab,cl,kw AND review*:ti,ab,cl,kw) OR ((meta:ti,ab,cl,kw OR meta-:ti,ab,cl,kw) AND analys*:ti,ab,cl,kw) OR (evidence:ti,ab,cl,kw AND synthes*:ti,ab,cl,kw) | 265,984 |
| 4 | Filter | 'animal'/exp NOT 'human'/exp | 542,758 |
| 5 | Search | #1 AND #2 AND #3 | 2,717 |
| 6 | Search | #5 NOT #4 | 2,678 |

**Epistemonikos- First systematic search**

**Researcher: Sophie Michel**

**URL:****https://www.epistemonikos.org/en/advanced_search**

**Date: 09. December 2020**

**Filters: from 01/01/95-current, Publication type= Systematic reviews**

| # | Concept | Search string | # of hits |
| --- | --- | --- | --- |
| 1 | Exposure | (title:(air pollut*) OR abstract:(air pollut*)) OR (title:(air toxin*) OR abstract:(air toxin*)) OR (title:(air toxic*) OR abstract:(air toxic*)) OR (title:(air quality) OR abstract:(air quality)) OR (title:(particulate matter) OR abstract:(particulate matter)) OR (title:(particle*) OR abstract:(particle*)) OR (title:(ultrafine) OR abstract:(ultrafine)) OR (title:(PM2.5) OR abstract:(PM2.5)) OR (title:(PM10) OR abstract:(PM10)) OR (title:(exhaust gas*) OR abstract:(exhaust gas*)) OR (title:(traffic-related pollut*) OR abstract:(traffic-related pollut*)) OR (title:(smog) OR abstract:(smog)) OR (title:(soot) OR abstract:(soot)) OR (title:(dust) OR abstract:(dust)) OR (title:(ozone) OR abstract:(ozone)) OR (title:(carbon monoxide*) OR abstract:(carbon monoxide*)) OR (title:(sulfur dioxide*) OR abstract:(sulfur dioxide*)) OR (title:(sulfurous anhydride*) OR abstract:(sulfurous anhydride*)) OR (title:(nitrogen oxide*) OR abstract:(nitrogen oxide*)) OR (title:(nitrogen dioxide*) OR abstract:(nitrogen dioxide*)) OR (title:(nitric oxide*) OR abstract:(nitric oxide*)) OR (title:(NO2) OR abstract:(NO2)) OR (title:(NOx) OR abstract:(NOx)) OR (title:(polycyclic aromatic hydrocarbon*) OR abstract:(polycyclic aromatic hydrocarbon*)) OR (title:(polynuclear aromatic hydrocarbon*) OR abstract:(polynuclear aromatic hydrocarbon*)) OR (title:(radon) OR abstract:(radon)) OR (title:(benzene*) OR abstract:(benzene*)) OR (title:(toluene*) OR abstract:(toluene*)) OR (title:(ethlybenzene*) OR abstract:(ethlybenzene*)) OR (title:(xylene*) OR abstract:(xylene*)) OR (title:(BTEX) OR abstract:(BTEX)) OR (title:(trichloroethane*) OR abstract:(trichloroethane*)) OR (title:(organic solvent*) OR abstract:(organic solvent*)) OR (title:(chlorinated solvent*) OR abstract:(chlorinated solvent*)) OR (title:(indoor air pollut*) OR abstract:(indoor air pollut*)) OR (title:(household air pollut*) OR abstract:(household air pollut*)) OR (title:(wood*) OR abstract:(wood*)) OR (title:(stove*) OR abstract:(stove*)) OR (title:(kitchen) OR abstract:(kitchen)) OR (title:(vent*) OR abstract:(vent*)) OR (title:(combust*) OR abstract:(combust*)) OR (title:(burn*) OR abstract:(burn*)) OR (title:(fuel*) OR abstract:(fuel*)) OR (title:(biofuel*) OR abstract:(biofuel*)) OR (title:(biogas) OR abstract:(biogas)) OR (title:(biodiesel) OR abstract:(biodiesel)) OR (title:(kerosene) OR abstract:(kerosene)) OR (title:(biomass) OR abstract:(biomass)) OR (title:(coal*) OR abstract:(coal*)) OR (title:(charcoal*) OR abstract:(charcoal*)) OR (title:(dung) OR abstract:(dung)) | 13,722 |
| 2 | Population | (title:(pregnan*) OR abstract:(pregnan*)) OR (title:(gestation) OR abstract:(gestation)) OR (title:(prenatal) OR abstract:(prenatal)) OR (title:(antenatal) OR abstract:(antenatal)) OR (title:(embryo*) OR abstract:(embryo*)) OR (title:(fetus) OR abstract:(fetus)) OR (title:(fetal) OR abstract:(fetal)) OR (title:(maternal exposure) OR abstract:(maternal exposure)) OR (title:(prenatal exposure delayed effect) OR abstract:(prenatal exposure delayed effect)) OR (title:(parturition) OR abstract:(parturition)) OR (title:(birth*) OR abstract:(birth*)) OR (title:(preterm) OR abstract:(preterm)) OR (title:(premature) OR abstract:(premature)) OR (title:(newborn*) OR abstract:(newborn*)) OR (title:(neonat*) OR abstract:(neonat*)) OR (title:(infant*) OR abstract:(infant*)) OR (title:(infancy) OR abstract:(infancy)) OR (title:(baby) OR abstract:(baby)) OR (title:(babies) OR abstract:(babies)) OR (title:(toddler*) OR abstract:(toddler*)) OR (title:(child*) OR abstract:(child*)) OR (title:(school) OR abstract:(school)) OR (title:(kindergar*) OR abstract:(kindergar*)) OR (title:(boys) OR abstract:(boys)) OR (title:(girls) OR abstract:(girls)) OR (title:(nursery) OR abstract:(nursery)) OR (title:(daycare) OR abstract:(daycare)) OR (title:(teenage*) OR abstract:(teenage*)) OR (title:(adolescen*) OR abstract:(adolescen*)) OR (title:(pubert*) OR abstract:(pubert*)) OR (title:(pubescen*) OR abstract:(pubescen*)) OR (title:(student*) OR abstract:(student*)) OR (title:(youth) OR abstract:(youth)) OR (title:(pediatric*) OR abstract:(pediatric*)) OR (title:(paediatric*) OR abstract:(paediatric*)) | 169,230 |
| 3 | Search | #1 AND #2 | 2,347 |

**Epistemonikos- Final updated systematic search**

**Researcher: Sophie Michel**

**URL:****https://www.epistemonikos.org/en/advanced_search**

**Date: 07. April 2023**

**Filters: from 01/01/2020-current, Publication type= Systematic reviews**

| # | Concept | Search string | # of hits |
| --- | --- | --- | --- |
| 1 | Exposure | (title:(air pollut*) OR abstract:(air pollut*)) OR (title:(air toxin*) OR abstract:(air toxin*)) OR (title:(air toxic*) OR abstract:(air toxic*)) OR (title:(air quality) OR abstract:(air quality)) OR (title:(particulate matter) OR abstract:(particulate matter)) OR (title:(particle*) OR abstract:(particle*)) OR (title:(ultrafine) OR abstract:(ultrafine)) OR (title:(PM2.5) OR abstract:(PM2.5)) OR (title:(PM10) OR abstract:(PM10)) OR (title:(exhaust gas*) OR abstract:(exhaust gas*)) OR (title:(traffic-related pollut*) OR abstract:(traffic-related pollut*)) OR (title:(smog) OR abstract:(smog)) OR (title:(soot) OR abstract:(soot)) OR (title:(dust) OR abstract:(dust)) OR (title:(ozone) OR abstract:(ozone)) OR (title:(carbon monoxide*) OR abstract:(carbon monoxide*)) OR (title:(sulfur dioxide*) OR abstract:(sulfur dioxide*)) OR (title:(sulfurous anhydride*) OR abstract:(sulfurous anhydride*)) OR (title:(nitrogen oxide*) OR abstract:(nitrogen oxide*)) OR (title:(nitrogen dioxide*) OR abstract:(nitrogen dioxide*)) OR (title:(NO2) OR abstract:(NO2)) OR (title:(NOx) OR abstract:(NOx)) OR (title:(polycyclic aromatic hydrocarbon*) OR abstract:(polycyclic aromatic hydrocarbon*)) OR (title:(polynuclear aromatic hydrocarbon*) OR abstract:(polynuclear aromatic hydrocarbon*)) OR (title:(radon) OR abstract:(radon)) OR (title:(benzene*) OR abstract:(benzene*)) OR (title:(toluene*) OR abstract:(toluene*)) OR (title:(ethlybenzene*) OR abstract:(ethlybenzene*)) OR (title:(xylene*) OR abstract:(xylene*)) OR (title:(BTEX) OR abstract:(BTEX)) OR (title:(trichloroethane*) OR abstract:(trichloroethane*)) OR (title:(organic solvent*) OR abstract:(organic solvent*)) OR (title:(chlorinated solvent*) OR abstract:(chlorinated solvent*)) OR (title:(indoor air pollut*) OR abstract:(indoor air pollut*)) OR (title:(household air pollut*) OR abstract:(household air pollut*)) OR (title:(wood*) OR abstract:(wood*)) OR (title:(stove*) OR abstract:(stove*)) OR (title:(kitchen) OR abstract:(kitchen)) OR (title:(vent*) OR abstract:(vent*)) OR (title:(combust*) OR abstract:(combust*)) OR (title:(burn*) OR abstract:(burn*)) OR (title:(fuel*) OR abstract:(fuel*)) OR (title:(biofuel*) OR abstract:(biofuel*)) OR (title:(biogas) OR abstract:(biogas)) OR (title:(biodiesel) OR abstract:(biodiesel)) OR (title:(kerosene) OR abstract:(kerosene)) OR (title:(biomass) OR abstract:(biomass)) OR (title:(coal*) OR abstract:(coal*)) OR (title:(charcoal*) OR abstract:(charcoal*)) OR (title:(dung) OR abstract:(dung)) | 42,677 |
| 2 | Population | (title:(pregnan*) OR abstract:(pregnan*)) OR (title:(gestation) OR abstract:(gestation)) OR (title:(prenatal) OR abstract:(prenatal)) OR (title:(antenatal) OR abstract:(antenatal)) OR (title:(embryo*) OR abstract:(embryo*)) OR (title:(fetus) OR abstract:(fetus)) OR (title:(fetal) OR abstract:(fetal)) OR (title:(maternal exposure) OR abstract:(maternal exposure)) OR (title:(prenatal exposure delayed effect) OR abstract:(prenatal exposure delayed effect)) OR (title:(parturition) OR abstract:(parturition)) OR (title:(birth*) OR abstract:(birth*)) OR (title:(preterm) OR abstract:(preterm)) OR (title:(premature) OR abstract:(premature)) OR (title:(newborn*) OR abstract:(newborn*)) OR (title:(neonat*) OR abstract:(neonat*)) OR (title:(infant*) OR abstract:(infant*)) OR (title:(infancy) OR abstract:(infancy)) OR (title:(baby) OR abstract:(baby)) OR (title:(babies) OR abstract:(babies)) OR (title:(toddler*) OR abstract:(toddler*)) OR (title:(child*) OR abstract:(child*)) OR (title:(school) OR abstract:(school)) OR (title:(kindergar*) OR abstract:(kindergar*)) OR (title:(boys) OR abstract:(boys)) OR (title:(girls) OR abstract:(girls)) OR (title:(nursery) OR abstract:(nursery)) OR (title:(daycare) OR abstract:(daycare)) OR (title:(teenage*) OR abstract:(teenage*)) OR (title:(adolescen*) OR abstract:(adolescen*)) OR (title:(pubert*) OR abstract:(pubert*)) OR (title:(pubescen*) OR abstract:(pubescen*)) OR (title:(student*) OR abstract:(student*)) OR (title:(youth) OR abstract:(youth)) OR (title:(pediatric*) OR abstract:(pediatric*)) OR (title:(paediatric*) OR abstract:(paediatric*)) | 137,125 |
| 3 | Search | #1 AND #2, Filter: Publication type= Systematic reviews | 7 |

Material S3: Risk of bias assessment results for each systematic review


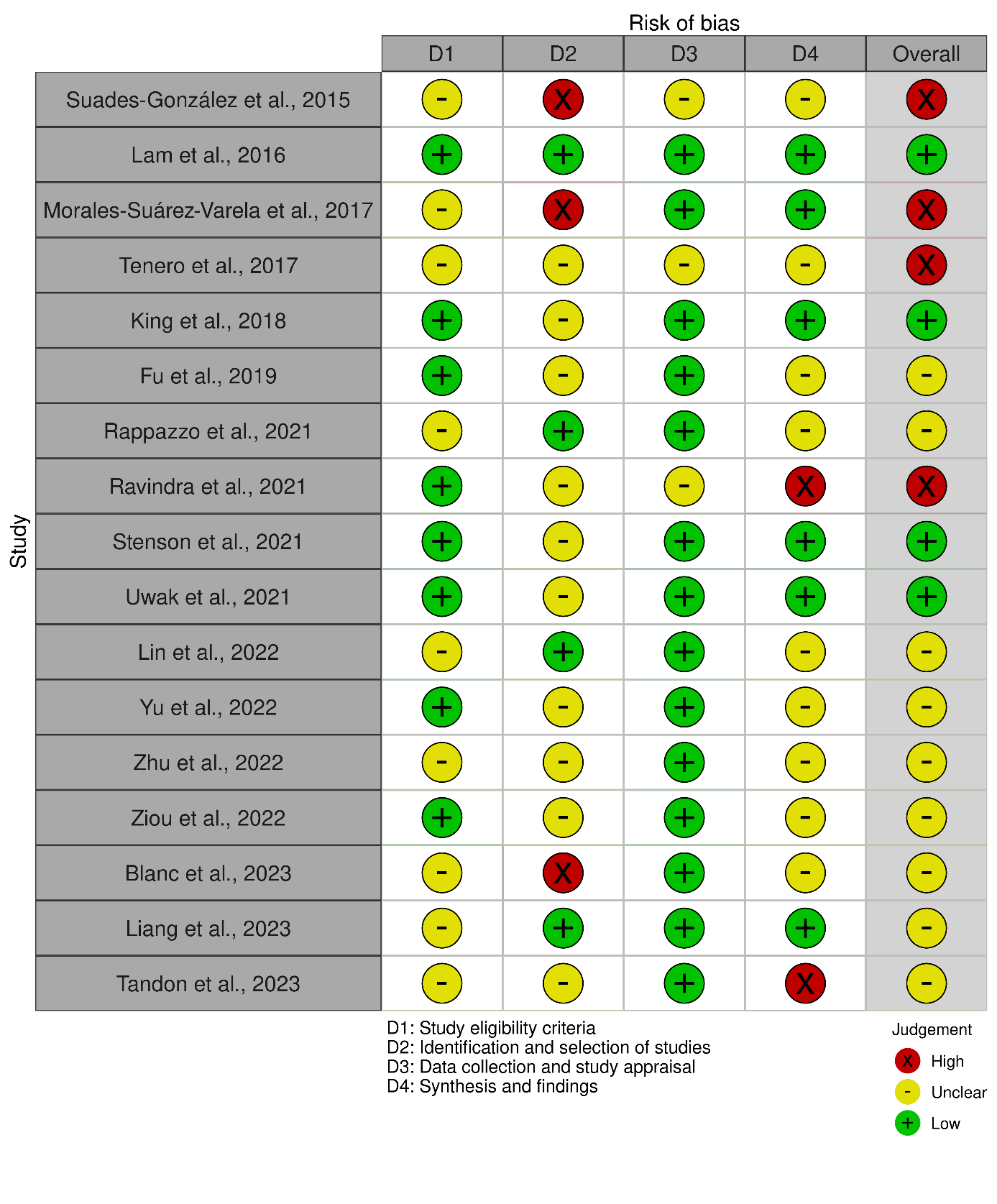


Figure S1: Risk of bias assessment results of each systematic review

Material S4: Responses to each question of the ROBIS tool

|  |  |  | **Suades-González et al., 2015** | **Lam et al., 2016** | **Morales-Suárez-Varela et al., 2017** | **Tenero et al., 2017** | **King et al., 2018** | **Fu et al., 2019** | **Rappazzo et al., 2021** | **Ravindra et al., 2021** | **Stenson et al., 2021** | **Uwak et al., 2021** | **Lin et al., 2022** | **Yu et al., 2022** | **Zhu et al., 2022** | **Ziou et al., 2022** | **Blanc et al., 2023** | **Liang et al., 2023** | **Tandon et al., 2023** |
| --- | --- | --- | --- | --- | --- | --- | --- | --- | --- | --- | --- | --- | --- | --- | --- | --- | --- | --- | --- |
| **DOMAIN 1: STUDY ELIGIBILITY CRITERIA** | | | Y/PY/PN/N/NI | Y/PY/PN/N/NI | Y/PY/PN/N/NI | Y/PY/PN/N/NI | Y/PY/PN/N/NI | Y/PY/PN/N/NI | Y/PY/PN/N/NI | Y/PY/PN/N/NI | Y/PY/PN/N/NI | Y/PY/PN/N/NI | Y/PY/PN/N/NI | Y/PY/PN/N/NI | Y/PY/PN/N/NI | Y/PY/PN/N/NI | Y/PY/PN/N/NI | Y/PY/PN/N/NI | Y/PY/PN/N/NI |
| 1.1 Did the review adhere to pre-defined objectives and eligibility criteria? | | | NI | Y | NI | NI | Y | NI | NI | NI | Y | Y | Y | Y | NI | Y | Y | NI | PY |
| 1.2 Were the eligibility criteria appropriate for the review question? | | | Y | Y | PY | PY | Y | Y | Y | Y | Y | Y | Y | Y | Y | Y | Y | Y | Y |
| 1.3 Were eligibility criteria unambiguous? | | | PN | Y | PY | PN | PY | PY | Y | PY | Y | Y | PY | Y | Y | Y | PY | Y | PY |
| 1.4 Were any restrictions in eligibility criteria based on study characteristics appropriate (e.g. date, sample size, study quality, outcomes measured)? | | | PY | Y | PY | Y | Y | Y | PY | Y | Y | Y | Y | Y | Y | Y | Y | PY | PY |
| 1.5 Were any restrictions in eligibility criteria based on sources of information appropriate (e.g. publication status or format, language, availability of data)? | | | PN | Y | PN | PN | PY | PN | PN | PN | PN | PN | N | PN | N | PN | PN | PN | NI |
| Concerns regarding specification of study eligibility criteria | | | UNCLEAR | LOW | UNCLEAR | UNCLEAR | LOW | LOW | UNCLEAR | LOW | LOW | LOW | UNCLEAR | LOW | UNCLEAR | LOW | UNCLEAR | UNCLEAR | UNCLEAR |
| **DOMAIN 2: IDENTIFICATION AND SELECTION OF STUDIES** | | |  |  |  |  |  |  |  |  |  |  |  |  |  |  |  |  |  |
| 2.1 Did the search include an appropriate range of databases/electronic sources for published and unpublished reports? | | | Y | Y | N | Y | Y | Y | Y | Y | Y | Y | Y | Y | Y | Y | Y | Y | Y |
| 2.2 Were methods additional to database searching used to identify relevant reports? | | | N | Y | N | Y | N | Y | Y | Y | Y | Y | Y | N | Y | N | N | Y | Y |
| 2.3 Were the terms and structure of the search strategy likely to retrieve as many eligible studies as possible? | | | NI | Y | NI | N | Y | NI | PY | PN | Y | PY | Y | Y | NI | Y | PN | Y | PY |
| 2.4 Were restrictions based on date, publication format, or language appropriate? | | | N | Y | NI | N | Y | NI | PY | N | NI | PN | PN | NI | NI | NI | PN | Y | NI |
| 2.5 Were efforts made to minimise error in selection of studies? | | | Y | Y | NI | Y | Y | Y | Y | NI | Y | Y | Y | Y | Y | Y | Y | Y | PY |
| Concerns regarding methods used to identify and/or select studies | | | HIGH | LOW | HIGH | UNCLEAR | UNCLEAR | UNCLEAR | LOW | UNCLEAR | UNCLEAR | UNCLEAR | LOW | UNCLEAR | UNCLEAR | UNCLEAR | HIGH | LOW | UNCLEAR |
| **DOMAIN 3: DATA COLLECTION AND STUDY APPRAISAL** | | |  |  |  |  |  |  |  |  |  |  |  |  |  |  |  |  |  |
| 3.1 Were efforts made to minimise error in data collection? | | | NI | Y | NI | NI | Y | Y | Y | Y | Y | Y | Y | Y | Y | Y | Y | Y | PY |
| 3.2 Were sufficient study characteristics available for both review authors and readers to be able to interpret the results? | | | PY | Y | Y | PY | Y | Y | Y | PN | Y | Y | Y | Y | PN | PY | Y | Y | Y |
| 3.3 Were all relevant study results collected for use in the synthesis? | | | PN | Y | Y | PN | Y | Y | PY | PY | Y | Y | Y | Y | Y | Y | Y | Y | Y |
| 3.4 Was risk of bias (or methodological quality) formally assessed using appropriate criteria? | | | PY | Y | Y | PY | Y | PY | PY | Y | Y | Y | Y | Y | Y | Y | Y | Y | Y |
| 3.5 Were efforts made to minimise error in risk of bias assessment? | | | NI | Y | Y | NI | Y | NI | Y | Y | Y | Y | Y | Y | NI | Y | Y | Y | PY |
| Concerns regarding methods used to collect data and appraise studies | | | UNCLEAR | LOW | LOW | UNCLEAR | LOW | LOW | LOW | UNCLEAR | LOW | LOW | LOW | LOW | LOW | LOW | LOW | LOW | LOW |
| **DOMAIN 4: SYNTHESIS AND FINDINGS** | | |  |  |  |  |  |  |  |  |  |  |  |  |  |  |  |  |  |
| 4.1 Did the synthesis include all studies that it should? | | | Y | Y | Y | Y | Y | Y | Y | Y | Y | Y | PY | Y | NI | Y | Y | Y | Y |
| 4.2 Were all pre-defined analyses reported or departures explained? | | | NI | Y | PY | NI | Y | PY | NI | NI | Y | Y | Y | Y | PY | Y | Y | PY | PY |
| 4.3 Was the synthesis appropriate given the nature and similarity in the research questions, study designs and outcomes across included studies? | | | PY | Y | PY | PY | Y | Y | PN | N | Y | Y | PN | PN | PN | Y | PY | Y | PN |
| 4.4 Was between-study variation (heterogeneity) minimal or addressed in the synthesis? | | | PY | Y | PY | PY | Y | PN | PN | PY | Y | PY | PN | PN | PN | PN | PY | PY | PY |
| 4.5 Were the findings robust, e.g. as demonstrated through funnel plot or sensitivity analyses? | | | PY | PY | PY | PY | PY | PN | PN | PN | PY | PY | PY | PY | PN | PY | PN | PY | PN |
| 4.6 Were biases in primary studies minimal or addressed in the synthesis? | | | PY | Y | PY | NI | PY | Y | PY | PY | PY | Y | Y | PY | PY | PY | Y | Y | PY |
| Concerns regarding the synthesis and findings | | | UNCLEAR | LOW | LOW | UNCLEAR | LOW | UNCLEAR | UNCLEAR | HIGH | LOW | LOW | UNCLEAR | UNCLEAR | UNCLEAR | UNCLEAR | UNCLEAR | LOW | HIGH |
| **RISK OF BIAS IN THE REVIEW** | | |  |  |  |  |  |  |  |  |  |  |  |  |  |  |  |  |  |
| A. Did the interpretation of findings address all of the concerns identified in Domains 1 to 4? | | | PN | Y | PY | PN | PY | PN | PN | N | PY | PY | PN | PN | PN | PN | PN | PY | N |
| B. Was the relevance of identified studies to the review's research question appropriately considered? | | | Y | Y | PY | PY | PY | Y | Y | PY | Y | Y | Y | Y | Y | Y | PY | Y | Y |
| C. Did the reviewers avoid emphasizing results on the basis of their statistical significance? | | | Y | Y | PY | PN | Y | Y | PY | PN | Y | PY | PN | PY | PY | PY | PN | PN | Y |
| Risk of bias in the review | | | HIGH | LOW | HIGH | HIGH | LOW | UNCLEAR | UNCLEAR | HIGH | LOW | LOW | UNCLEAR | UNCLEAR | UNCLEAR | UNCLEAR | UNCLEAR | UNCLEAR | UNCLEAR |

PN: probably no, PY: probably yes, N: No, NI: no information, Y: Yes
